# Effects of GLP-1 Receptor Agonists on Biological Behavior of Colorectal Cancer Cells by Regulating PI3K/AKT/mTOR Signaling Pathway

**DOI:** 10.3389/fphar.2022.901559

**Published:** 2022-08-10

**Authors:** Guoxiang Tong, Tianhao Peng, Ya Chen, Lijuan Sha, Huikang Dai, Yidong Xiang, Zhiqi Zou, Heli He, Sha Wang

**Affiliations:** ^1^ Academician Workstation, Changsha Medical University, Changsha, China; ^2^ Department of Endocrinology, The First Affiliated Hospital of Changsha Medical University, Changsha, China; ^3^ Hunan Evidence-based Biotechnology Co., Ltd., Changsha, China; ^4^ Department of Oncology, The First Affiliated Hospital of Changsha Medical University, Changsha, China

**Keywords:** colorectal cancer, GLP-1 receptor agonist, liraglutide, PI3K/AKT/mTOR, migration, apoptosis

## Abstract

Colorectal cancer (CRC) has become one of the top ten malignant tumors with a high incidence rate and mortality. Due to the lack of a good CRC screening program, most of the CRC patients are being transferred at the time of treatment. The conventional treatment cannot effectively improve the prognosis of CRC patients, and the target drugs can significantly prolong the overall survival of patients in the advanced stage. However, the use of single drug may lead to acquired drug resistance and various serious complications. Therefore, combined targeted drug therapy is the main alternative treatment with poor effect of single targeted drug therapy, which has important research significance for the treatment of CRC. Therefore, this study intends to culture CRC cell lines *in vitro* at the cell level and intervene with the GLP-1 receptor agonist liraglutide. The effects of liraglutide on the PI3K/Akt/mTOR signal pathway and CRC cell proliferation, cycle, migration, invasion, and apoptosis are explored by detecting cell proliferation, cycle, migration, invasion, and apoptosis and the expression of related mRNA and protein. The results showed that liraglutide, a GLP-1 receptor agonist, could block the CRC cell cycle, reduce cell proliferation, migration, and invasion and promote apoptosis by inhibiting the PI3K/Akt/mTOR signal pathway.

## 1 Introduction

Colorectal cancer (CRC) refers to cancers that occur in the epithelium of the large intestine, including colon cancer and rectal cancer. Adenocarcinoma is the most common pathological type, and a few are squamous cell carcinoma. In China, rectal cancer is the most common type, followed by colon cancer, and the colon includes the sigmoid colon, cecum, ascending colon, descending colon, and transverse colon, which are widely affected. The incidence rate of CRC is high, and CRC has become one of the top ten malignant tumors (Brittain et al., 2018; Geijsen et al., 2019). In recent years, the incidence rate and mortality rate of CRC in China has been increasing obviously. Due to the lack of perfect CRC examination programs in China, most CRC patients are treated for metastasis, 15%–20% of CRC patients have liver metastasis in the initial operation, and liver metastasis recurrence 2 years after surgery is up to 50 (Peng et al., 2021). Based on the fact that conventional treatment methods cannot effectively improve the prognosis of CRC patients, molecular targeted drugs came into being. In addition to significantly prolonging the overall survival of patients with advanced stage, the high specificity of targeted drugs meets the requirements of current precision treatment. However, whether they are monoclonal antibodies or signal pathway inhibitors, except for patients with natural drug resistance, the use of single drug may lead to acquired drug resistance and various serious complications (Sadat et al., 2021; Teo et al., 2021; Huaiyu Zhang et al., 2021), making the treatment fail. Therefore, combined targeted drug therapy is the main alternative treatment with poor effect of single targeted drug therapy, which has important research significance for the treatment of CRC.

Glucagon-like peptide-1 (GLP-1) is widely distributed in the heart, brain, lung, gastrointestinal tract, pancreas, and other tissues. Activation of the GLP-1 receptor can promote a variety of cell proliferation and improve cell viability (Knudsen et al., 2018). Previous studies have shown that GLP-1 receptor agonists play an important role in the occurrence and development of a variety of tumor cells (Liu et al., 2019; Kojima et al., 2020; Mao et al., 2021). It has also been found that long-term injection of GLP-1 receptor agonists exenatide and liraglutide can promote the occurrence of thyroid cancer in rodents by activating the GLP-1 receptor in a dose-dependent manner (Sherman et al., 2018). GLP-1 receptor agonists have long-term effects on the occurrence and development of tumors originating from thyroid follicular cells (van den Brink et al., 2017). Therefore, GLP-1 receptor agonists may have a certain effect on tumor cells.

At present, academic circles believe that the accelerated progress of CRC is caused by PIK3CA gene mutation of the PI3K/Akt/mTOR signal pathway and the loss of expression of phosphatase and tensin homolog deleted on chromosome 10 (PTEN). PIK3CA mutation is closely related to the location, degree of tissue differentiation, and immunohistochemical type of CRC lesions (Zhihang Chen et al., 2020; Li et al., 2021; Wang et al., 2021; Jing Zhang et al., 2021). Cells carry out biological activities through proliferation, migration, invasion, and apoptosis, as well as cancer cells (Xie et al., 2021). So far, although there are many studies on CRC, the pathogenesis and pathophysiological mechanism of CRC are still not fully understood. Understanding and mastering the changes of CRC cell proliferation, migration, cycle, and apoptosis can provide an important theoretical basis for the research and treatment of CRC.

Based on the aforementioned points, the effects of GLP-1 receptor agonists on tumor cells, and the effects of the PI3K/Akt/mTOR signaling pathway on cell biology, we boldly put forward the scientific hypothesis that GLP-1 receptor agonists can affect the proliferation, cycle, migration, invasion, and apoptosis of CRC cells by mediating PI3K/Akt/mTOR. Therefore, this study intends to culture CRC cell lines *in vitro* at the cell level and intervene with the GLP-1 receptor agonist liraglutide. By detecting the changes in cell proliferation, cycle, migration, invasion, and apoptosis and the expression of related mRNAs and proteins, this study will explore the effects of liraglutide on the PI3K/Akt/mTOR signal pathway and CRC cell proliferation, cycle, migration, invasion, and apoptosis, and preliminarily explore its possible mechanisms of action so as to provide an experimental and theoretical basis for CRC, combined with targeted drug therapy.

## 2 Materials and Methods

### 2.1 Cell Culture and Passage

Human normal colonic epithelial cell line NCI-H661 was cultured in high-glucose DMEM containing 10% fetal bovine serum, and human colon cancer cell line LOVO was cultured in RPMI1640 medium in a sterile incubator at 37°C with 5% CO_2_ saturation humidity. When the cells reached 70%–80% confluence when observed under a microscope, they were digested and subcultured with 0.25% trypsin. Tumor cells in the logarithmic growth stage were taken for the experiment. Materials such as cells and culture medium were purchased from Beijing Dingguo Changsheng Biotechnology Co., Ltd.

### 2.2 Liraglutide Intervention and Grouping

LOVO cells in the logarithmic growth stage were inoculated into a 96-well culture plate with 5,000 cells/well, and the culture medium was 100 μ L/well, cultured in a sterile incubator at 37°C, 5% CO_2_ saturation humidity for 24 h, and then the supernatant was gently sucked and discarded. In order to study the optimal intervention concentration, liraglutide (Macklin) at different concentrations (10^−5 ^mol/L, 10^−8 ^mol/L, 10^−11 ^mol/L) was used to intervene. After 24, 48, and 72 h of intervention, 5 mg/ml MTT 20 μL was added to each well. It was incubated at 37°C for 4 h, the liquid in the hole was discarded, and DMSO 150 μL was added to each hole. After shaking for 10 min without light, the absorbance value (A value) of each hole was measured at 450 nm wavelength using the enzyme-linked immunosorbent assay instrument (Thermo Fisher Scientific). The intervention concentration with the highest inhibition rate of 10^−8^ mol/L was selected, and LOVO cells were randomly divided into three groups: 1) blank control group; 2) metformin (MedBio) control group: 20 mmol/L metformin; and 3) liraglutide group: 10^−8^ mol/L liraglutide.

### 2.3 Real-Time Fluorescence Quantitative PCR (qRT-PCR)

A certain amount of TRIzol solution (absin) was added to CRC cells to extract total RNA from the cells. After zeroing the micro-nucleic acid protein detector with 0.1% DEPC water, 1 μ L was used to detect the concentration and purity of the extracted RNA diluent. When 1.8 < A260/A280 < 2.0, it was indicated that the purity of the RNA was qualified and there was no pollution of protein and phenol. RNA concentration = 40 × A260ng/μ L. Then the reading was taken. RNA concentration at 500–1,000 ng/μ L room is the most suitable. An appropriate amount of 0.1% DEPC water was added for adjustment. The cDNA was synthesized using reverse transcription. The primers were purchased from Shanghai Jima. The primer sequence required for the reaction: GAPDH: F: 5 ′- tca​aga​tca​gca​atg​cc-3′, R: 5 ′- cga​tac​aca​agt​tgt​cat​gga-3'; PI3K: F:5′- CAG​ACA​GCG​AGA​GAG​ATG​A-3′, R:5′-GGTGGATGGTGGTGCTGTATT-3’; Akt: F:5′- ATT​GTG​AAG​GAG​GGT​TGG​CTG-3′, R:5′-CCGCTCCTTGTAGCCAATGAA -3’; mTOR: F:5′- CCC​TCC​ATC​CAC​CTC​ATC​AGT-3′, R:5′-CGCCAAGACACAGTAGCGAAT-3’. Pre-denaturation: 95°C 2min, amplification: 95 °C 5s, 60 °C 30s, melt curve program: 95 °C 15s, 60 °C 60s, 95 °C 10sec. The reverse transcription kit was purchased from Thermo Fisher. It was operated according to the user guide of the fluorescence quantitative kit, U6 and GAPDH were taken as internal parameters, and the relative content of PI3K/Akt/mTOR in CRC was calculated by using the 2^-△△Ct^ method (Wang et al., 2021).

### 2.4 Plate Cloning

Each group of cells in logarithmic growth stage was blown with 0.25% trypsin as a single cell, and the cells were suspended in the RPMI1640 medium containing 10% fetal bovine serum. The cell suspension was inoculated in a petri dish containing 10 ml of 37°C preheated medium, gently rotated to disperse the cells evenly, and placed in a sterile incubator with 37°C and 5% CO_2_ saturation humidity for 2–3 weeks. The culture medium was changed according to the cell growth. When the cells had clones visible to the naked eye, the culture was terminated, the supernatant was discarded, and the cells were cleaned and fixed. Then an appropriate amount of Giemsa was added and dyed with the staining solution for 30 min The staining solution was then cleaned and dried, and the cells were made into a transparent film. The number of clones greater than 10 cells under the microscope and the clone formation rate were calculated = (number of clones/number of inoculated cells) × 100%。

### 2.5 Wound Healing Experiment

LOVO cells in the logarithmic growth stage were taken and were divided into two cells per well × the density of 104 cells was inoculated on 96-well plates and incubated for 24 h, and scratches were made on the confluent adherent cell layer using wound healing inserts. The medium was sucked out, and the cells were gently washed with the medium twice. Then the plate was placed inside the imaging system, and scanning was repeated for 24 h. Calculation of mobility: mobility = (1–24 h scratch distance/initial distance) × 100%.

### 2.6 Transwell

Matrigel (Corning) was dissolved at 4°C overnight 2 days in advance and was diluted in serum-free medium in the ratio of 1:7. All operations involving Matrigel needed to be carried out on ice. The Matrigel invasion chamber was placed in a sterile 24-well plate, and 20 μl of Matrigel was added to the chamber so that it was evenly spread on the bottom of the chamber. CRC cells in each group were prepared into single cell suspension in serum-free RPMI1640 medium and counted under the microscope (the concentration is about 60–80 × 10^4^/ml). Medium 600 μl containing 10% fetal bovine serum was added to the small holes of the lower chamber, and 105 cells were added to each hole of the upper chamber to supplement 200 μl with serum-free medium. The cells were cultured at 37°C in an incubator with 5% carbon dioxide volume fraction for 24 h. When the cells had been worn for enough time, the upper chamber culture medium was discarded, the upper chamber was taken out carefully, and the cells not passing through the membrane were gently wiped with a wet cotton swab. The chamber was washed with PBS 3 times, and the lower part of the chamber was fixed with 4% paraformaldehyde (Beijing Dingguo Changsheng Biotechnology Co., Ltd.) for 30 min, dyed with crystal violet for 20 min. Photos were taken under an inverted microscope. Randomly five visual fields were chosen, and the number of cells penetrating the stromal membrane was counted.

### 2.7 Flow Cytometry

Cells were washed twice with pre-chilled PBS, then washed, and resuspended with 1× binding buffer to adjust the cell concentration to 1×10^6^ cells/mL; 100 μL of the adjusted cell suspension was taken into 5 mL of flow cytometry tube. Annexin V-labeled protein and corresponding nucleic acid dyes were added, then mixed evenly, and kept at room temperature for 15 min; 400 μL of 1× binding buffer was added to the flow cytometry tube, resuspended, and analyzed by using a flow cytometer in time. After the flow cytometer is installed, the data will be directly analyzed, and the time of reading the data will not be recorded.

### 2.8 Western Blotting

Total protein in CRC cells was extracted, and the BCA protein quantification kit was used for protein quantification and analysis. Protein sample weighing 50 μg was taken, added to the sample well of 5% stacking gel, and 3 μl protein marker was added to the sample well, and the total amount added to each well was equal. After identifying the positive and negative electrodes of the electrophoresis tank, electrophoresis was started. The initial constant voltage was 90 V. When the bottom of the bromophenol blue reached the surface of the separation gel, the voltage was adjusted to 120 V, and the electrophoresis was run until the bromophenol blue dye ran out of the separation gel. The electrophoresis instrument was turned off. The glass plate was gently removed, the stacking gel and excess separating gel were cut off according to the Marker strip, and the gel containing the target protein was soaked in transfer buffer. The membrane was transferred, blocked, and incubated with primary antibody: the PVDF membrane from the blocking solution was taken out, and the membrane was washed three times with TBST solution, 10 min/time. The primary antibodies (rabbit anti-human PI3K, 1:1,000; Akt, 1:1,000; mTOR, 1:1,000; cyclin D1, 1:1,000; MMP-11, 1:1,000) diluted with TBST were added and incubated at 4°C overnight. Reaction with secondary antibody: the PVDF membrane from the primary antibody was taken out and washed 3 times in TBST, 5 min/time. The IR fluorescently labeled secondary antibody (diluted with TBST) was added and incubated for 2 h in the dark on a shaker (pay attention to moderate speed). This procedure was repeated for the TBST cells 3 times, 5 min/time, and in the dark. The Odyssey dual-color infrared fluorescence scanning system was used to observe the colored bands, image, and analyze the data.

### 2.9 Statistical Analysis

The data of this study were processed by SPSS 19.0 software for statistical analysis. Measurement data were expressed as mean ± standard deviation. The comparison of means between groups was performed using one-way ANOVA, the comparison of data between two groups was performed using the *t*-test of two independent samples, and the comparison within two groups was performed using the SNK-q test. *p* < 0.05 was considered to be statistically significant.

## 3 Results

### 3.1 Expression of PI3K/Akt/mTOR in Colorectal Cancer Cells

**FIGURE 1:**
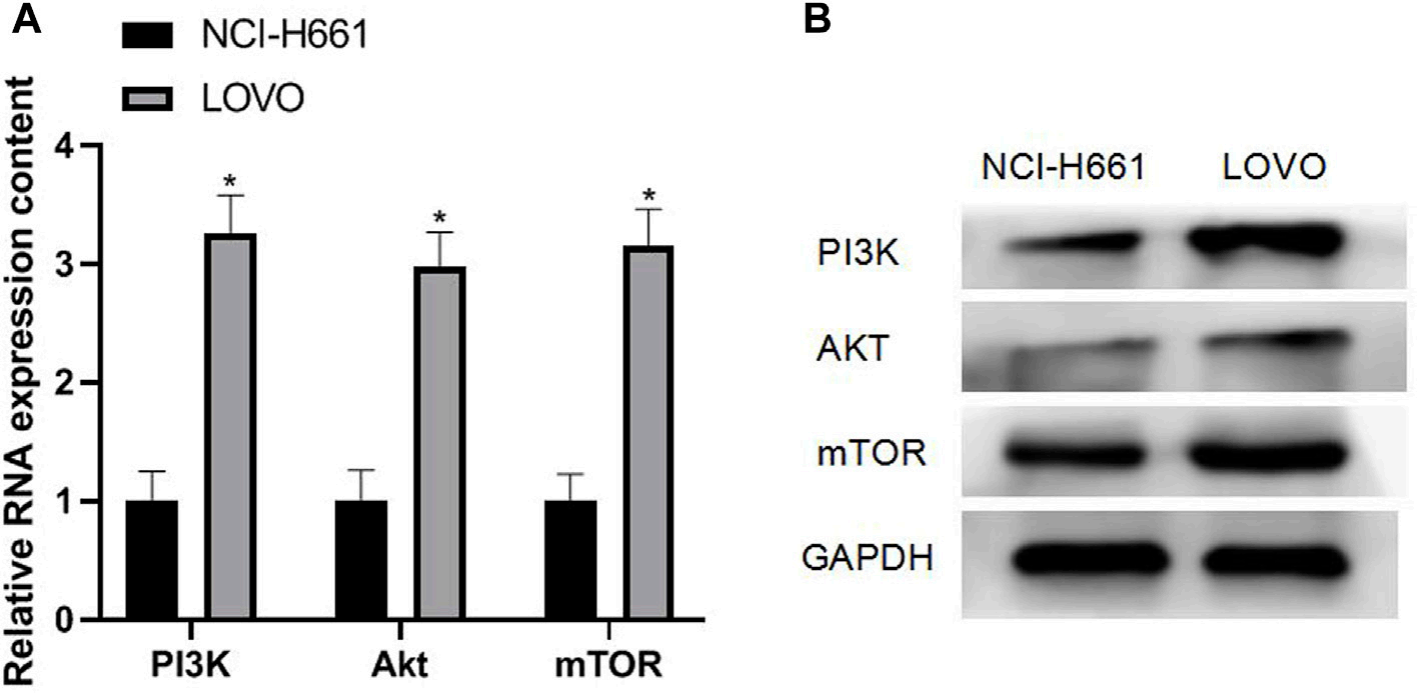
Expression of PI3K/Akt/mTOR in CRC cells. Note: **(A)** qRT-PCR experiment; **(B)** Western blotting experiment, compared with NCI-H661, *p < 0.05.

The results of qRT-PCR and Western blotting experiments showed that the mRNA and protein expressions of PI3K (3.26 ± 0.32), Akt (2.98 ± 0.29), and mTOR (3.16 ± 0.30) were all upregulated in CRC cells, and the difference was statistically significant (*p* < 0.05). As shown in Figures 1A,B

### 3.2 Effects of GLP-1 Receptor Agonists on the Proliferation and Cycle of Colorectal Cancer Cells

**FIGURE 2:**
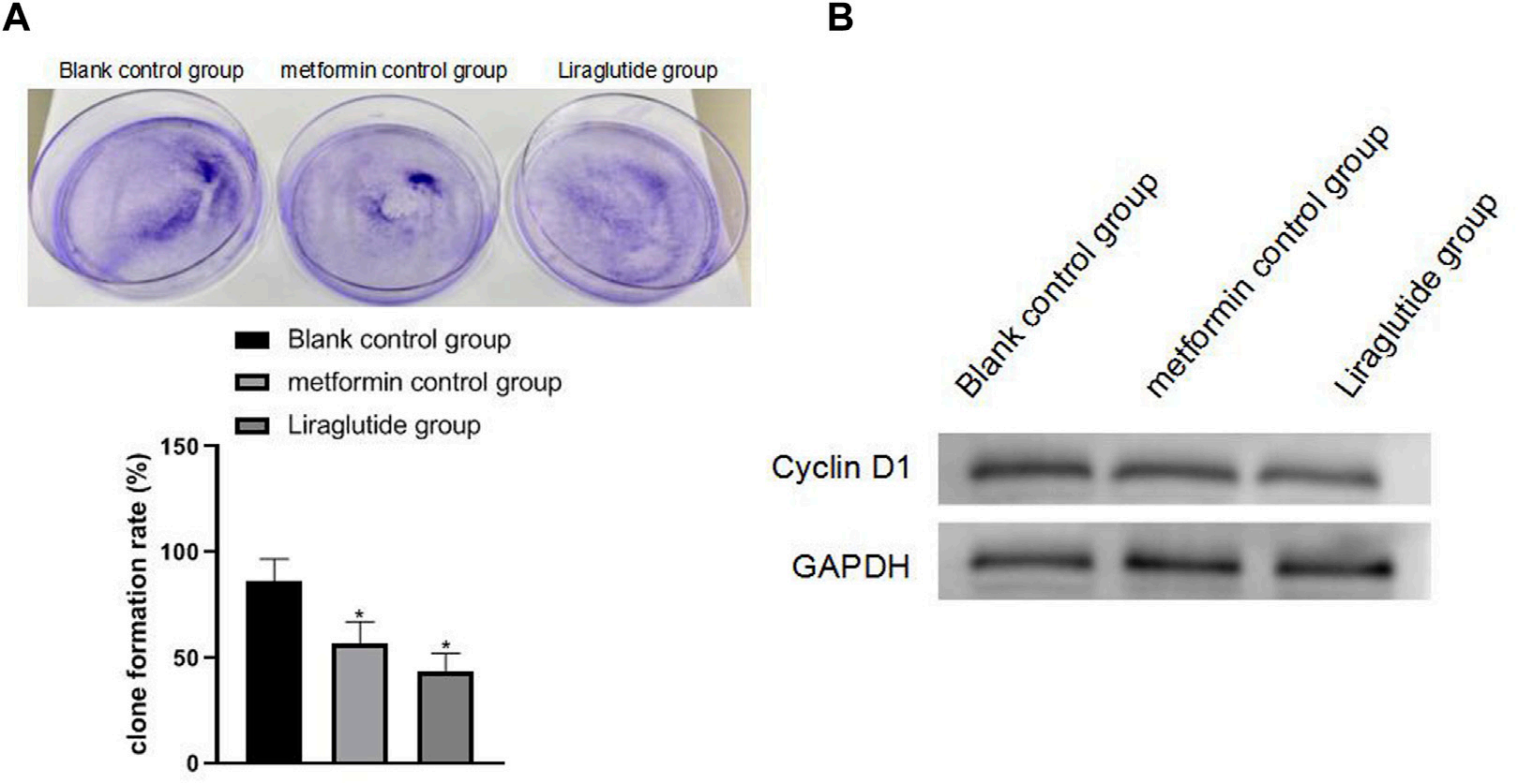
Effects of GLP-1 receptor agonists on the proliferation and cycle of CRC cells. Note: **(A)** plate cloning experiment; **(B)** Western blotting experiment, compared with blank control group, *p < 0.05.

The results of plate cloning and Western blotting experiments showed that after the intervention of the GLP-1 receptor agonist liraglutide, the proliferation ability of CRC cells weakened (13.19 ± 8.85), the expression of cyclin D1 protein decreased, and the difference was statistically significant (*p* < 0.05), as shown in Figures 2A,B

### 3.3 Effects of GLP-1 Receptor Agonists on the Migration and Invasion of Colorectal Cancer Cells

**FIGURE 3:**
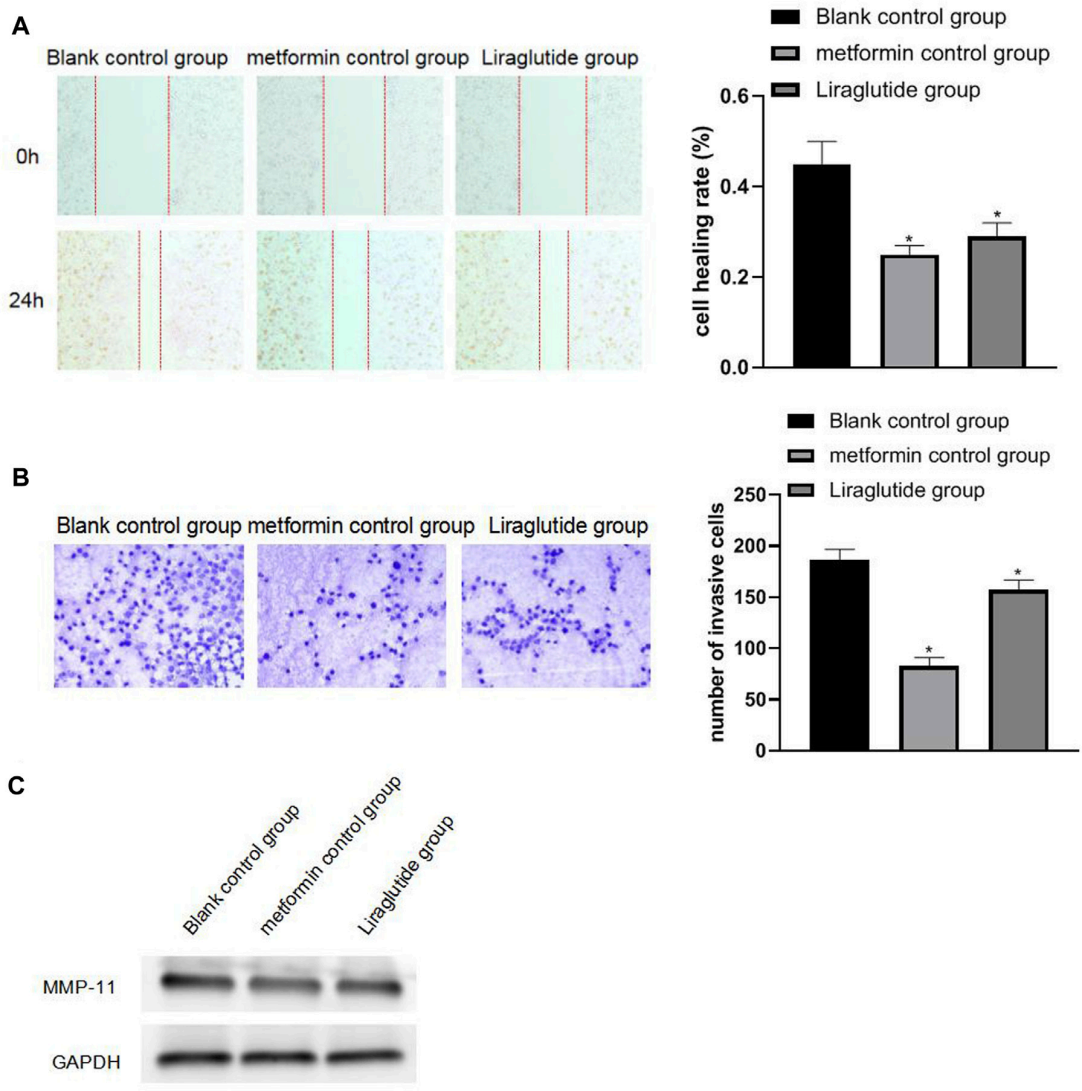
Effects of GLP-1 receptor agonists on the migration and invasiveness of CRC cells. Note: **(A)** wound healing experiment; **(B)** Transwell experiment (20 × ); **(C)** Western blotting experiment, compared with blank control group, *p < 0.05.

The results of wound healing, Transwell, and Western blotting experiments showed that after the intervention of the GLP-1 receptor agonist liraglutide, the migration (0.29 ± 0.03) and invasion ability (157.11 ± 9.66) of CRC cells weakened, the expression of MMP-11 protein was decreased, and the differences were statistically significant (*p* < 0.05), as shown in Figures 3A,B,C.

### 3.4 Effects of GLP-1 Receptor Agonists on Apoptosis of Colorectal Cancer Cells

**FIGURE 4:**
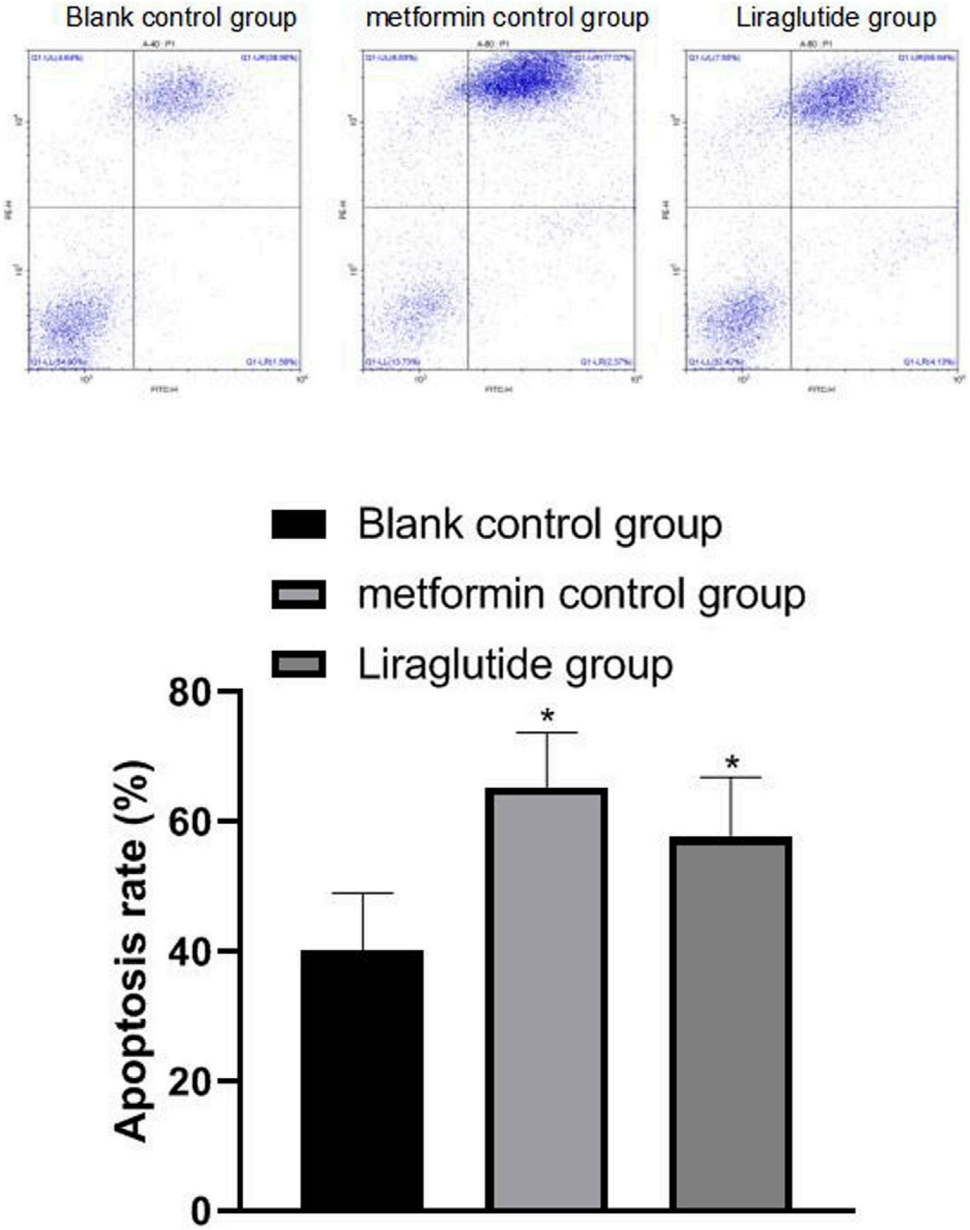
Effects of GLP-1 receptor agonists on apoptosis of CRC cells. Note: flow cytometry experiment, compared with blank control group, *p < 0.05.

The results of flow cytometry showed that after the intervention of the GLP-1 receptor agonist liraglutide, the apoptosis (57.69 ± 9.21) of CRC cells increased and the difference was statistically significant (*p* < 0.05), as shown in Figure 4.

### 3.5 Effects of GLP-1 Receptor Agonists on the Expression of PI3K/Akt/mTOR in Colorectal Cancer Cells

**FIGURE 5:**
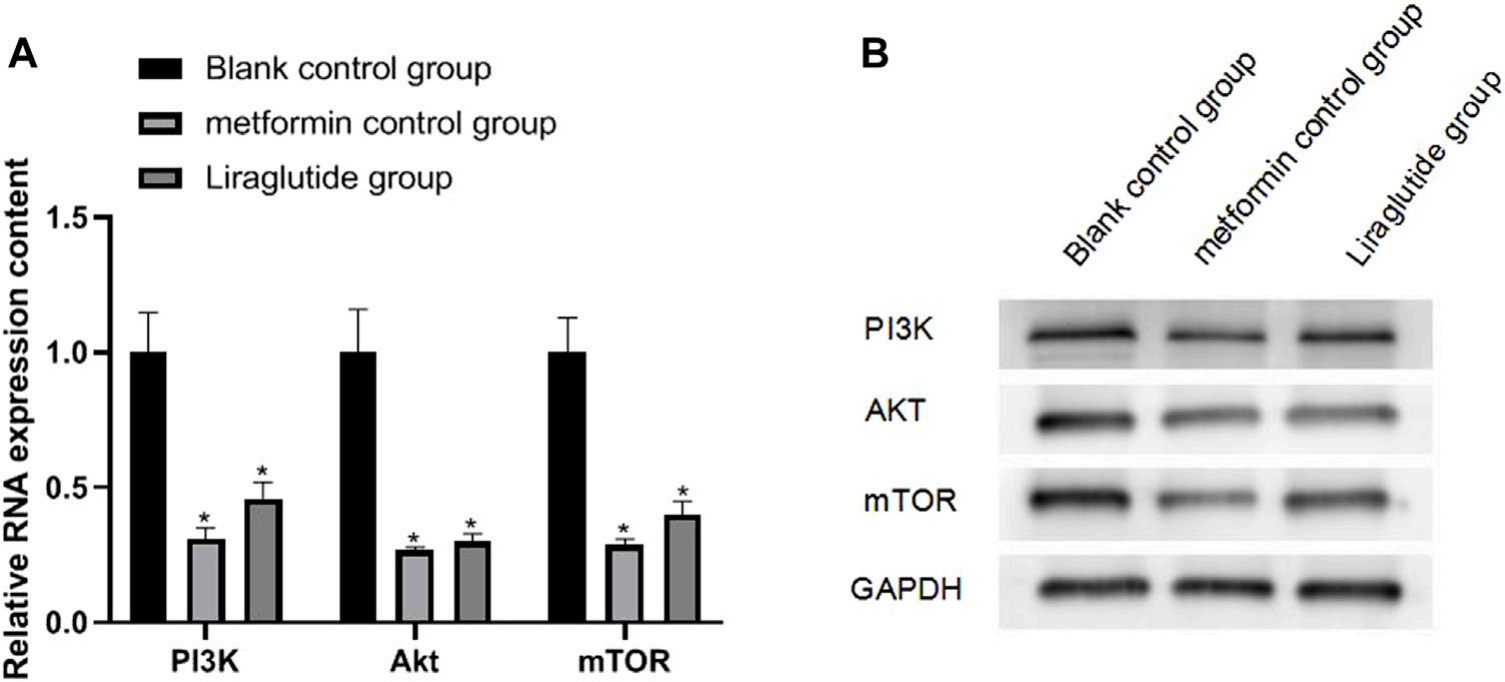
Effects of GLP-1 receptor agonists on the expression of PI3K/Akt/mTOR in CRC cells. Note: **(A)** qRT-PCR experiment; **(B)** Western blotting experiment, compared with blank control group, *p < 0.05.

The results of qRT-PCR and Western blotting experiments showed that after the intervention of the GLP-1 receptor agonist liraglutide, the expressions of PI3K (0.46 ± 0.06), Akt (0.30 ± 0.03), and mTOR (0.40 ± 0.05) proteins in CRC cells were decreased, and the difference was statistically significant (*p* < 0.05), as shown in Figure 5A,B.

## 4 Discussion

According to the GLOBOCAN project of the WHO Cancer Research Center, the number of new cases of CRC in 2018 was about 1.8 million, and the number of deaths was about 880,000. The incidence of CRC is related to factors such as age, region, and gender (Takiyama et al., 2017; Zhou et al., 2022). The pathological types of CRC are divided into ulcerative, raised, and infiltrating. (Reid et al., 2021). In the early stage of CRC, there are no obvious symptoms, and some non-specific symptoms such as changes in bowel habits and changes in stool characteristics may appear, which are easy to be ignored. However, as the disease progressed, various gastrointestinal symptoms and systemic symptoms, such as blood in the stool and abdominal pain were seen (Zhang and Jiang, 2020). Conventional treatment methods cannot effectively improved the prognosis of CRC patients, and targeted drugs can significantly prolong the overall survival of advanced patients. However, the use of a single drug may lead to acquired drug resistance and various serious complications. Therefore, it is of great significance to explore new treatment methods for CRC.

Studies have shown that the activation of the PI3K/Akt/mTOR pathway begins with the activation of PIK3CA by the transmembrane tyrosine kinase growth factor receptor, which then activates Akt through phosphorylation and dephosphorylation reactions, which stimulate tuberous sclerosis 1 (TSC1). The TSC1/TSC2 complex generates Ras protein brain tissue homologous analogs, and the Ras protein brain tissue homologous analogs activate mTOR and then, respectively, controls the translation of specific subsets of messenger RNAs, thereby regulating protein synthesis and playing an important role in cell proliferation, invasion, metastasis, and metabolism (Huiqin Chen et al., 2020; Liu et al., 2021). This study found that in CRC cells, the expressions of PI3K, Akt, and mTOR were upregulated, indicating that the PI3K/Akt/mTOR pathway was activated, which is consistent with the aforementioned theory. Studies have shown that PIK3CA mutation can reduce the risk of CRC peritoneal metastasis (Lund-Andersen et al., 2021). Mutated BRAF, PIK3CA, and PTEN have also been confirmed to exist in the EGFR signal transduction pathway. Multi-site gene mutations lead to the complexity of tumor signal transduction, which explains the use of EGFR monoclonal antibodies in the treatment of CRC patients. The reason is that the prolongation of time and the time of disease progression of the patients did not form a significant promotion relationship, and because of this, the process of single-molecule targeted drugs as first-line drugs was gradually prolonged (Klupp et al., 2021). In addition, hypoxia-inducible factor-1α acts as a downstream regulatory unit of mTORC1, and its regulation of protein level is affected not only by the environmental oxygen concentration but also by interfering with the expression of hypoxia-inducible factor-1α in the PI3K/Akt signaling pathway, affecting the proliferation activity of CRC cells, thereby reducing the drug resistance of CRC cells (Deng et al., 2021), which further proves the necessity of in-depth research on PI3K/Akt/mTOR signaling pathway inhibitors.

GLP-1 receptor agonists act on islet beta cells in a glucose-dependent manner, promote insulin gene transcription, and inhibit islet beta cell apoptosis, thereby increasing the number of islet beta cells. The contents are emptied (Falkentoft et al., 2022). Zhao et al. (2014) studies confirm that GLP-1 receptor activation can inhibit the growth of human pancreatic cancer cells and promote cell apoptosis in a cAMP-dependent manner. Koehler et al. (2011) showed that GLP-1 receptor agonists can inhibit the growth and apoptosis of mouse CT26 colon cancer cells. Ligumsky et al.’s (2012) studies confirm that GLP-1 receptor agonists can inhibit the growth of breast cancer cells by activating cAMP (Hicks et al., 2016). Studies have shown that GLP-1 receptor agonists can mediate the signal pathway of phosphatidylinositol-3-kinase (PI3K)/protein kinase B (PKB/Akt)/mammalian target of rapamycin (mTOR), which has a protective effect on cerebral ischemia, but there are few studies on the role of PI3K/Akt/mTOR in CRC (Wang et al., 2019).

In the following elaboration, we explain that the cell cycle affects cell proliferation and thus correlates with the likelihood of cancer cell proliferation and spread. Therefore, it is necessary to block the growth and metastasis of cancer cells to curb the spread of cancer. After the intervention of the GLP-1 receptor agonist liraglutide, it was found that the proliferation, migration, and invasion of CRC cells weakened, cyclin D1 and MMP-11 decreased, and apoptosis increased. Cyclin D1 is an important protein in the cell cycle. As the regulator of cyclin-dependent kinase CDKs, cyclin D1 affects cell proliferation by regulating the transition from G1 the phase to S phase in the cell proliferation cycle (Cao et al., 2018; El-Gendi and Abu-Sheasha, 2018). MMP-11 is one of the matrix metalloproteinases (MMPs), which is the main enzyme causing the degradation of the basement membrane and extracellular matrix. Different family members of MMP may play a role in the occurrence and development of different tumors and are closely related to the low degree of tumor differentiation and strong ability of invasion and metastasis. Apoptotic pathways include the extracellular pathway and intracellular pathway. The expression of cyclin D1 and MMP-11 decreased, the cycle of cancer cells was blocked, and the metastatic ability of cancer cells was inhibited.

In conclusion, the PI3K/Akt/mTOR signaling pathway is activated in CRC. After intervention with a GLP-1 receptor agonist, CRC cells can block the cell cycle, inhibit cell proliferation, migration and invasion, promote apoptosis, and reduce the expression of PI3K/Akt/mTOR pathway protein. Therefore, GLP-1 receptor agonists can reduce the development of biological behavior of CRC cells by inhibiting the PI3K/Akt/mTOR signaling pathway.

## Data Availability

The data presented in the study are deposited in the repository of Hunan Evidence-Based Biotechnology Co., Ltd. (enterprise email, login number: hnxzsw888@vip.163.com).

## References

[B1] Brittain K. Pennings Kamp K. J. Salaysay Z. (2018). Colorectal Cancer Awareness for Women via Facebook: A Pilot Study. Gastroenterol. Nurs. 41 (1), 14–18. 10.1097/SGA.0000000000000294 29373351 PMC6040828

[B2] Cao L. Liu Y. Wang D. Huang L. Li F. Liu J. (2018). MiR-760 Suppresses Human Colorectal Cancer Growth by Targeting BATF3/AP-1/cyclinD1 Signaling. J. Exp. Clin. Cancer Res. 37 (1), 83. 10.1186/s13046-018-0757-8 29661228 PMC5902951

[B3] Chen H. Chen N. Li F. Sun L. Du J. Chen Y. (2020). Repeated Radon Exposure Induced Lung Injury and Epithelial-Mesenchymal Transition through the PI3K/AKT/mTOR Pathway in Human Bronchial Epithelial Cells and Mice. Toxicol. Lett. 334, 4–13. 10.1016/j.toxlet.2020.09.008 32949624

[B4] Chen Z. Wang C. Dong H. Wang X. Gao F. Zhang S. (2020). Aspirin Has a Better Effect on PIK3CA Mutant Colorectal Cancer Cells by PI3K/Akt/Raptor Pathway. Mol. Med. 26 (1), 14. 10.1186/s10020-020-0139-5 32000660 PMC6993447

[B5] Deng X. Kong F. Li S. Jiang H. Dong L. Xu X. (2021). A KLF4/PiHL/EZH2/HMGA2 Regulatory axis and its Function in Promoting Oxaliplatin-Resistance of Colorectal Cancer. Cell Death Dis. 12 (5), 485. 10.1038/s41419-021-03753-1 33986248 PMC8119946

[B6] El-Gendi S. Abu-Sheasha G. (2018). Ki-67 and Cell Cycle Regulators P53, P63 and cyclinD1 as Prognostic Markers for Recurrence/Progression of Bladder Urothelial Carcinoma. Pathol. Oncol. Res. 24 (2), 309–322. 10.1007/s12253-017-0250-2 28488128

[B7] Falkentoft A. C. Andersen J. Malik M. E. Selmer C. Gæde P. H. Staehr P. B. (2022). Impact of Socioeconomic Position on Initiation of SGLT-2 Inhibitors or GLP-1 Receptor Agonists in Patients with Type 2 Diabetes - a Danish Nationwide Observational Study. Lancet Reg. Health Eur. 14, 100308. 10.1016/j.lanepe.2022.100308 35146474 PMC8802041

[B8] Geijsen A. J. M. R. Brezina S. Keski-Rahkonen P. Baierl A. Bachleitner-Hofmann T. Bergmann M. M. (2019). Plasma Metabolites Associated with Colorectal Cancer: A Discovery-Replication Strategy. Int. J. Cancer 145 (5), 1221–1231. 10.1002/ijc.32146 30665271 PMC6614008

[B9] Hicks B. M. Yin H. Yu O. H. Pollak M. N. Platt R. W. Azoulay L. (2016). Glucagon-like Peptide-1 Analogues and Risk of Breast Cancer in Women with Type 2 Diabetes: Population Based Cohort Study Using the UK Clinical Practice Research Datalink. BMJ 355, i5340. 10.1136/bmj.i5340 27797785

[B10] Klupp F. Sass M. Bergmann F. Khajeh E. Ghamarnejad O. Hassenpflug M. (2021). Impact of EGFR and EGFR Ligand Expression on Treatment Response in Patients with Metastatic Colorectal Cancer. Oncol. Lett. 21 (6), 448. 10.3892/ol.2021.12709 33868486 PMC8045155

[B11] Knudsen J. S. Thomsen R. W. Pottegård A. Knop F. K. Sørensen H. T. (2018). Differences between Randomized Clinical Trial Patients and Real-World Initiators of the Glucagon-like Peptide 1 Receptor Agonist Liraglutide. Diabetes Care 41 (9), e133–e135. 10.2337/dc18-0999 30002200

[B12] Koehler J. A. Kain T. Drucker D. J. (2011). Glucagon-like Peptide-1 Receptor Activation Inhibits Growth and Augments Apoptosis in Murine CT26 colon Cancer Cells. Endocrinology 152 (9), 3362–3372. 10.1210/en.2011-1201 21771884

[B13] Kojima M. Takahashi H. Kuwashiro T. Tanaka K. Mori H. Ozaki I. (2020). Glucagon-Like Peptide-1 Receptor Agonist Prevented the Progression of Hepatocellular Carcinoma in a Mouse Model of Nonalcoholic Steatohepatitis. Int. J. Mol. Sci. 21 (16), 5722. 10.3390/ijms21165722 PMC7460814 32785012

[B14] Li H. Huang H. Li S. Mei H. Cao T. Lu Q. (2021). Long Non-Coding RNA ADAMTS9-AS2 Inhibits Liver Cancer Cell Proliferation, Migration and Invasion. Exp. Ther. Med. 21 (6), 559. 10.3892/etm.2021.9991 33850531 PMC8027749

[B15] Ligumsky H. Wolf I. Israeli S. Haimsohn M. Ferber S. Karasik A. (2012). The Peptide-Hormone Glucagon-like Peptide-1 Activates cAMP and Inhibits Growth of Breast Cancer Cells. Breast Cancer Res. Treat. 132 (2), 449–461. 10.1007/s10549-011-1585-0 21638053

[B16] Liu Y. Zhang X. Chai S. Zhao X. Ji L. (2019). Risk of Malignant Neoplasia with Glucagon-like Peptide-1 Receptor Agonist Treatment in Patients with Type 2 Diabetes: A Meta-Analysis. J. Diabetes Res. 2019, 1534365. 10.1155/2019/1534365 31396537 PMC6664552

[B17] Liu Z. Sun T. Piao C. Zhang Z. Kong C. (2021). METTL13 Inhibits Progression of clear Cell Renal Cell Carcinoma with Repression on PI3K/AKT/mTOR/HIF-1α Pathway and C-Myc Expression. J. Transl Med. 19 (1), 209. 10.1186/s12967-021-02879-2 33985542 PMC8120818

[B18] Lund-Andersen C. Torgunrud A. Fleten K. G. Flatmark K. (2021). Omics Analyses in Peritoneal Metastasis-Utility in the Management of Peritoneal Metastases from Colorectal Cancer and Pseudomyxoma Peritonei: a Narrative Review. J. Gastrointest. Oncol. 12 (Suppl. 1), S191–S203. 10.21037/jgo-20-136 33968437 PMC8100703

[B19] Mao D. Cao H. Shi M. Wang C. C. Kwong J. Li J. J. X. (2021). Increased Co-expression of PSMA2 and GLP-1 Receptor in Cervical Cancer Models in Type 2 Diabetes Attenuated by Exendin-4: A Translational Case-Control Study. EBioMedicine 65, 103242. 10.1016/j.ebiom.2021.103242 33684886 PMC7938253

[B20] Peng Y. Feng H. Wang C. Song Z. Zhang Y. Liu K. (2021). The Role of E26 Transformation-specific Variant Transcription Factor 5 in Colorectal Cancer Cell Proliferation and Cell Cycle Progression. Cel Death Dis. 12 (5), 427. 10.1038/s41419-021-03717-5 PMC8087822 33931578

[B21] Reid F. S. W. Egoroff N. Pockney P. G. Smith S. R. (2021). A Systematic Scoping Review on Natural Killer Cell Function in Colorectal Cancer. Cancer Immunol. Immunother. 70 (3), 597–606. 10.1007/s00262-020-02721-6 32918127 PMC10992123

[B22] Sadat S. M. A. Paiva I. M. Shire Z. Sanaee F. Morgan T. D. R. Paladino M. (2021). A Synthetically Lethal Nanomedicine Delivering Novel Inhibitors of Polynucleotide Kinase 3'-phosphatase (PNKP) for Targeted Therapy of PTEN-Deficient Colorectal Cancer. J. Control. Release 334, 335–352. 10.1016/j.jconrel.2021.04.034 33933518

[B23] Sherman S. I. Kloos R. T. Tuttle R. M. Pontecorvi A. Völzke H. Harper K. (2018). No Calcitonin Change in a Person Taking Dulaglutide Diagnosed with Pre-existing Medullary Thyroid Cancer. Diabet Med. 35 (3), 381–385. 10.1111/dme.13437 28755389 PMC5838554

[B24] Takiyama A. Tanaka T. Yamamoto Y. Hata K. Ishihara S. Nozawa H. (2017). Microsatellite Status of Primary Colorectal Cancer Predicts the Incidence of Postoperative Colorectal Neoplasms. Anticancer Res. 37 (10), 5785–5790. 10.21873/anticanres.12020 28982902

[B25] Teo M. Y. M. Ng J. J. C. Fong J. Y. Hwang J. S. Song A. A. Lim R. L. H. (2021). Development of a Single-Chain Fragment Variable Fused-Mutant HALT-1 Recombinant Immunotoxin against G12V Mutated KRAS Colorectal Cancer Cells. PeerJ 9, e11063. 10.7717/peerj.11063 33959410 PMC8053384

[B26] van den Brink W. Emerenciana A. Bellanti F. Della Pasqua O. van der Laan J. W. (2017). Prediction of Thyroid C-Cell Carcinogenicity after Chronic Administration of GLP1-R Agonists in Rodents. Toxicol. Appl. Pharmacol. 320, 51–59. 10.1016/j.taap.2017.02.010 28213092

[B27] Wang J. Wang A. He H. She X. He Y. Li S. (2019). Trametenolic Acid B Protects against Cerebral Ischemia and Reperfusion Injury through Modulation of microRNA-10a and PI3K/Akt/mTOR Signaling Pathways. Biomed. Pharmacother. 112, 108692. 10.1016/j.biopha.2019.108692 30798122

[B28] Wang J. Zhou F. Li F. Wang B. Hu Y. Li X. (2021). Autocrined Leptin Promotes Proliferation of Non-small Cell Lung Cancer (NSCLC) via PI3K/AKT and P53 Pathways. Ann. Transl Med. 9 (7), 568. 10.21037/atm-20-7482 33987266 PMC8105803

[B29] Xie J. Sun Y. Xu Q. (2021). Inhibition of SRSF3 Alleviates Proliferation and Migration of Gastric Cancer Cells by Regulating the PI3K/AKT/mTOR Signalling Pathway. Folia Biol. (Praha) 67 (3), 102–107. 35151243 10.14712/fb2021067030102

[B30] Zhang H. Zhang J. Liu Y. Jiang Y. Li Z. (2021). Molecular Targeted Agent and Immune Checkpoint Inhibitor Co-Loaded Thermosensitive Hydrogel for Synergistic Therapy of Rectal Cancer. Front. Pharmacol. 12, 671611. 10.3389/fphar.2021.671611 33935796 PMC8085774

[B31] Zhang J. Hu J. Li W. Zhang C. Su P. Wang Y. (2021). Rapamycin Antagonizes BCRP-Mediated Drug Resistance through the PI3K/Akt/mTOR Signaling Pathway in mPRα-Positive Breast Cancer. Front. Oncol. 11, 608570. 10.3389/fonc.2021.608570 33912444 PMC8071953

[B32] Zhang W. Jiang K. W. (2020). Role of Gut Microbiota in Carcinogenesis and Treatment for Colorectal Cancer. Zhonghua Wei Chang Wai Ke Za Zhi 23 (5), 516–520. 10.3389/fphar.2021.671611 32842435

[B33] Zhao H. Wei R. Wang L. Tian Q. Tao M. Ke J. (2014). Activation of Glucagon-like Peptide-1 Receptor Inhibits Growth and Promotes Apoptosis of Human Pancreatic Cancer Cells in a cAMP-dependent Manner. Am. J. Physiol. Endocrinol. Metab. 306 (12), E1431–E1441. 10.1152/ajpendo.00017.2014 24801389

[B34] Zhou X. Wang L. Xiao J. Sun J. Yu L. Zhang H. (2022). Alcohol Consumption, DNA Methylation and Colorectal Cancer Risk: Results from Pooled Cohort Studies and Mendelian Randomization Analysis. Int. J. Cancer 1, 25. 10.1002/ijc.33945 PMC9487984 35102554

