# Supplementary figures and images for: Effects of GLP-1 Receptor Agonists on Biological Behavior of Colorectal Cancer Cells by Regulating PI3K/AKT/mTOR Signaling Pathway
Source: Front Pharmacol. 2022 Aug 10;13:901559. doi: 10.3389/fphar.2022.901559 (PMC9399678; doi:10.3389/fphar.2022.901559)

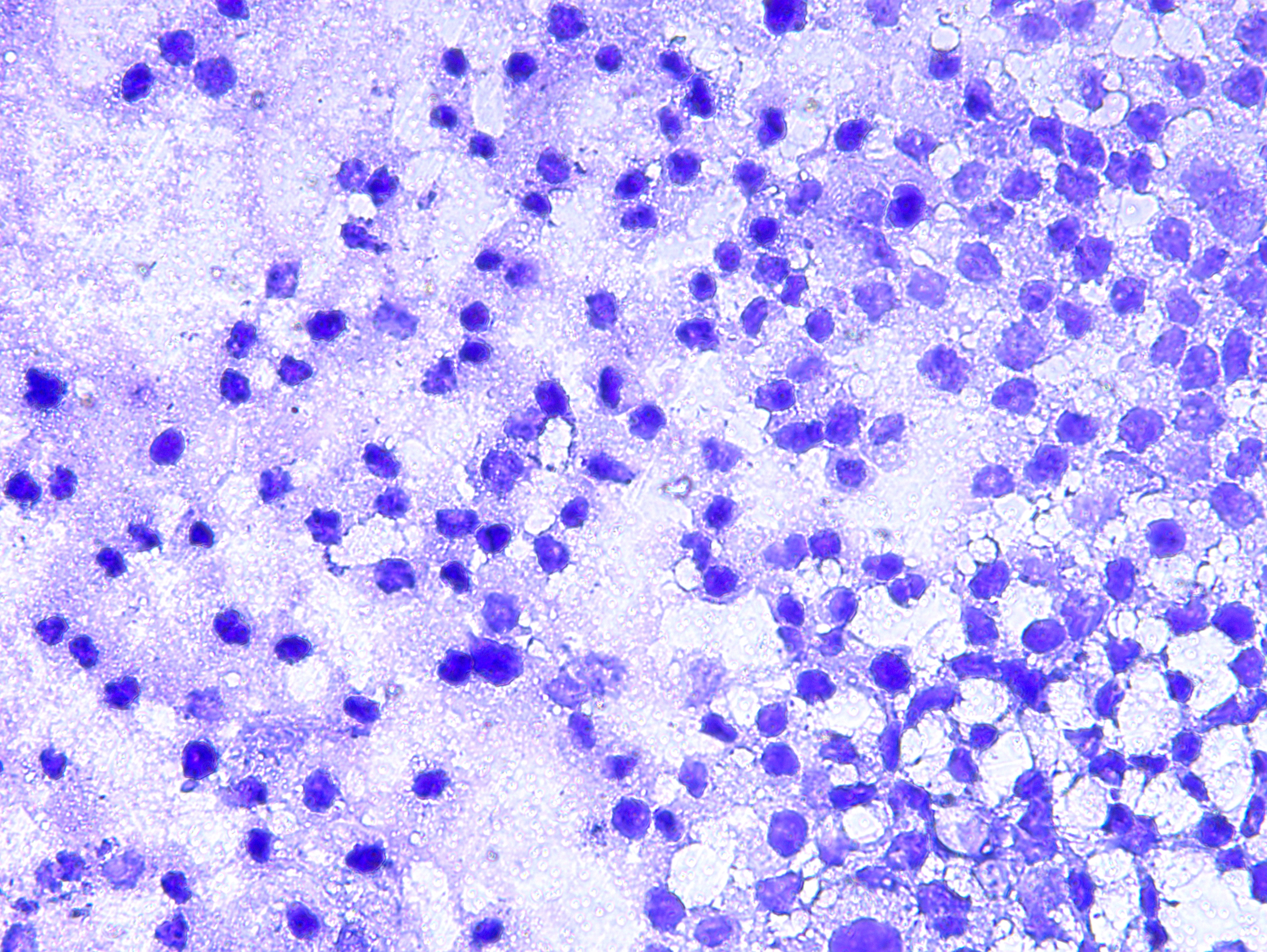

Supplement: Supplementary file 1 [file DataSheet3.ZIP › Transwell/Blank control group.jpg]

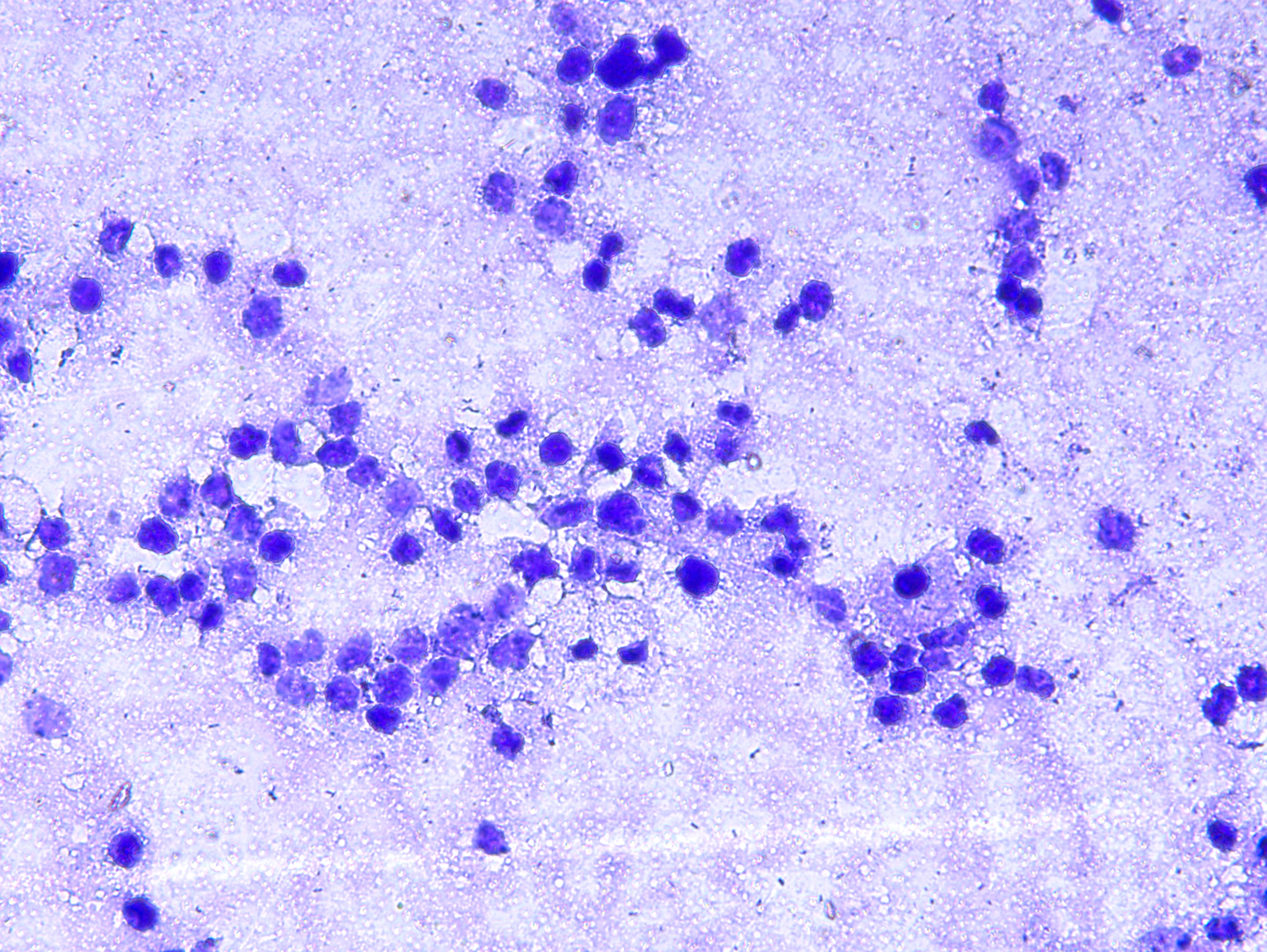

Supplement: Supplementary file 1 [file DataSheet3.ZIP › Transwell/Liraglutide group.jpg]

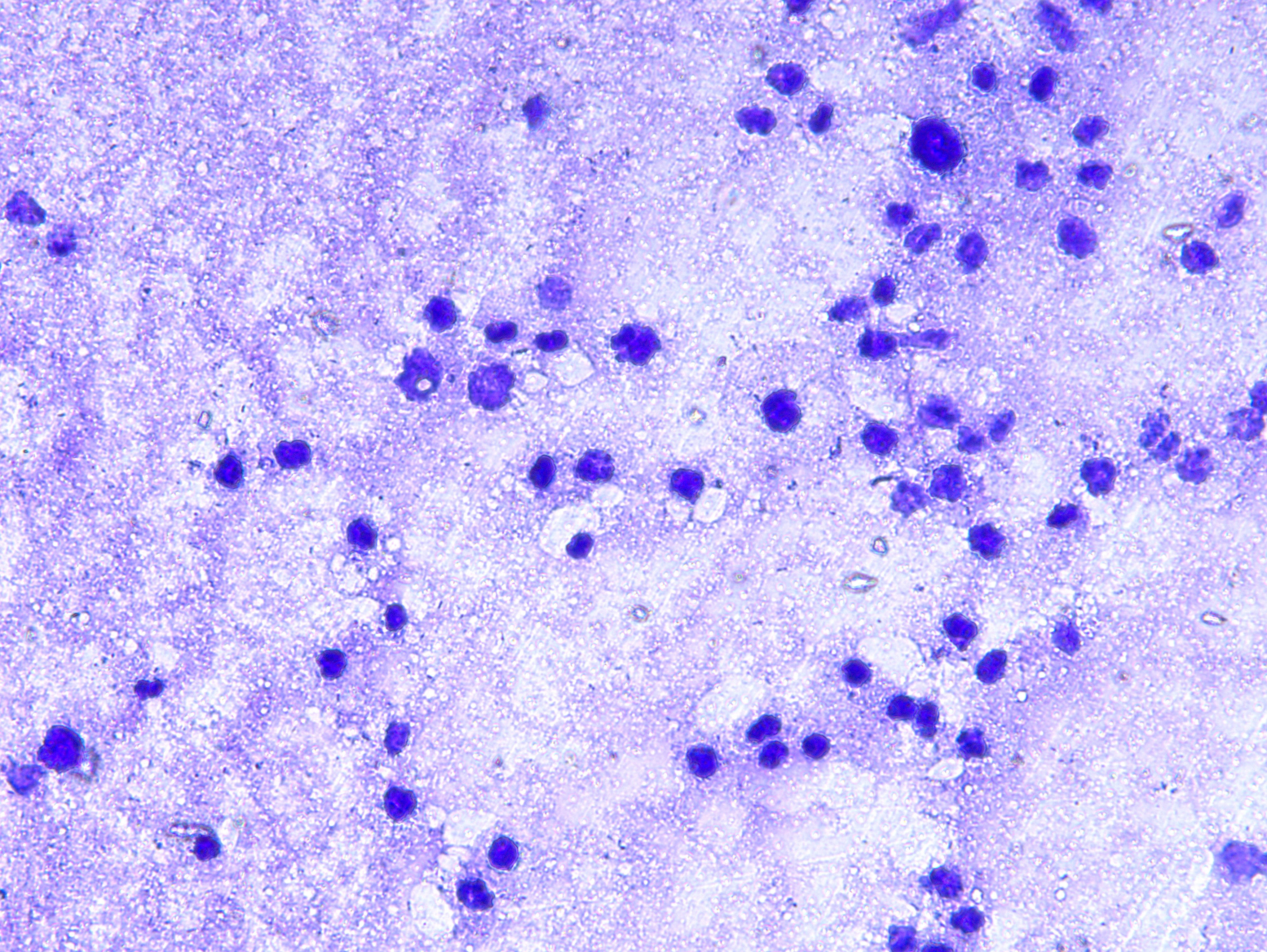

Supplement: Supplementary file 1 [file DataSheet3.ZIP › Transwell/metformin control group.jpg]

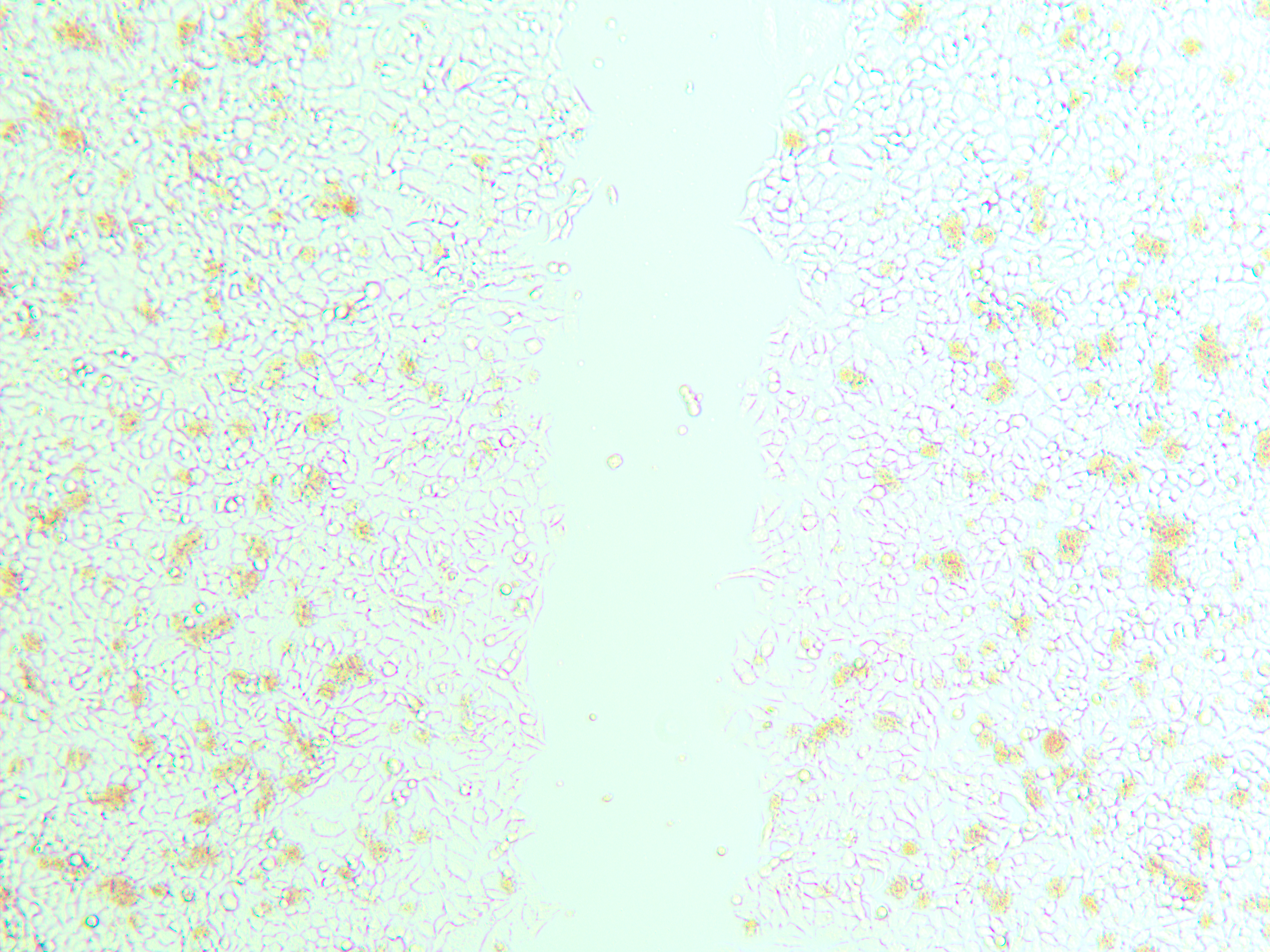

Supplement: Supplementary file 2 [file DataSheet8.ZIP › Liraglutide group12h.bmp]

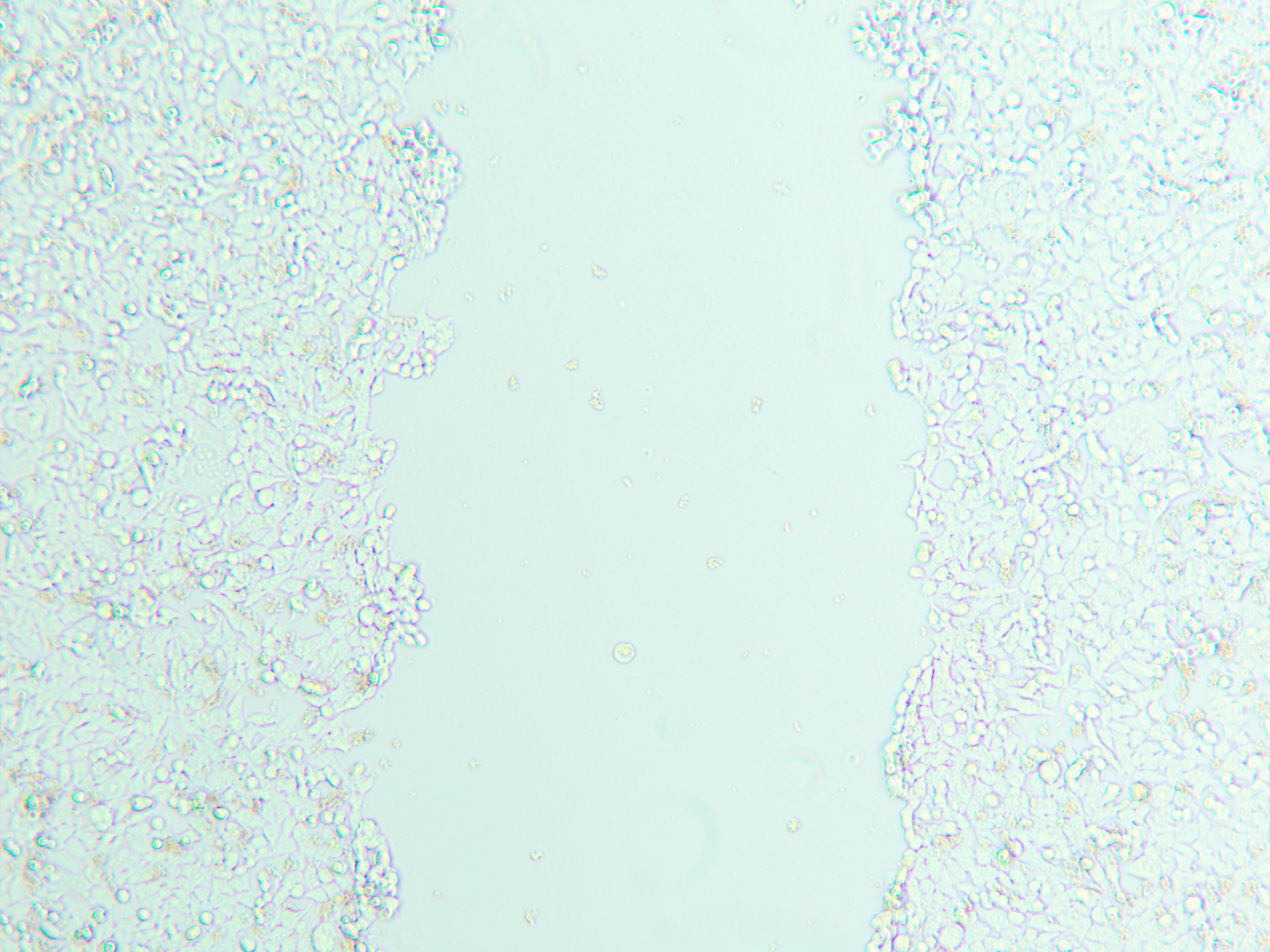

Supplement: Supplementary file 3 [file DataSheet9.ZIP › metformin control group 0h.bmp]

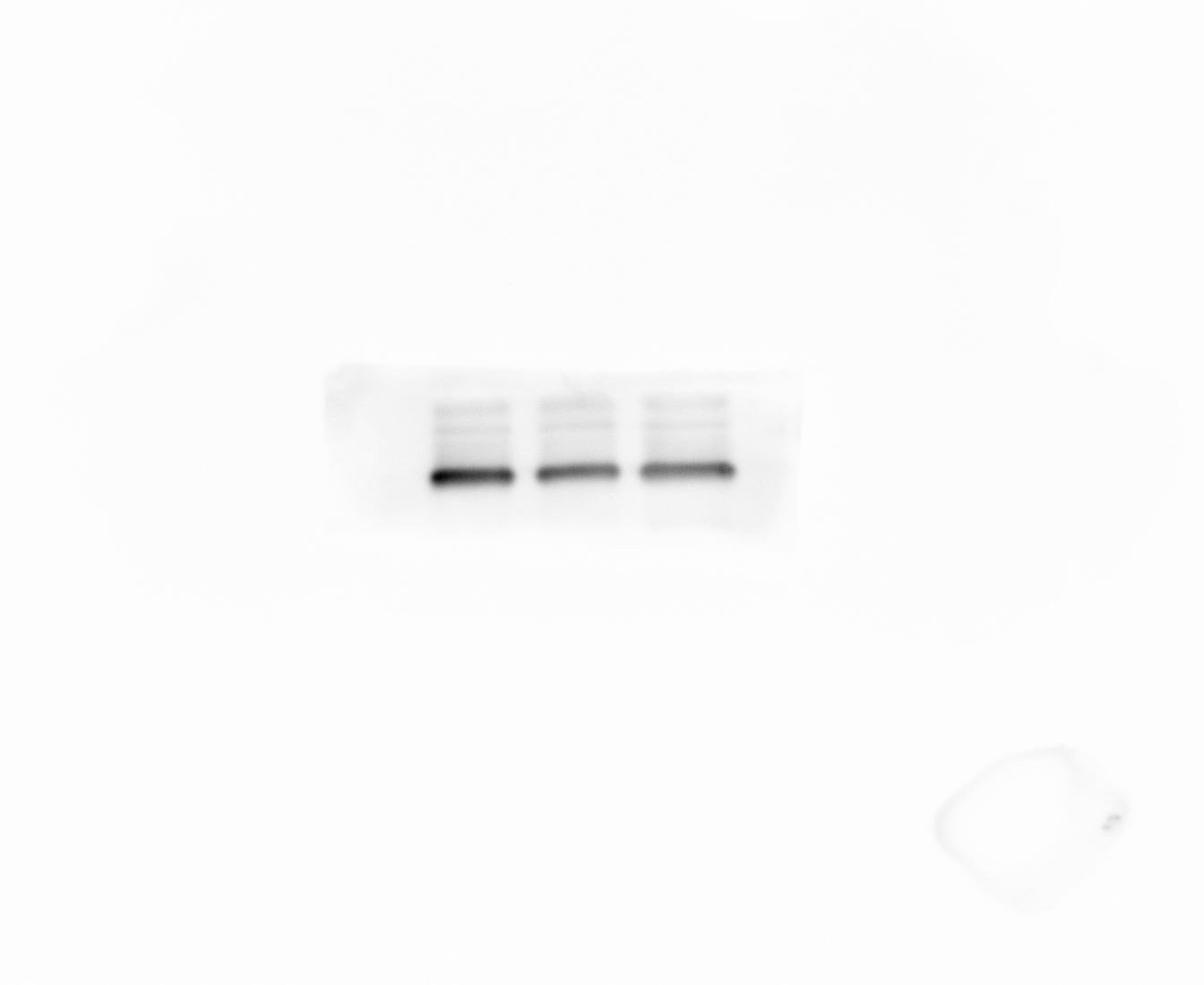

Supplement: Supplementary file 4 [file DataSheet4.ZIP › Western blot/AKt.tif]

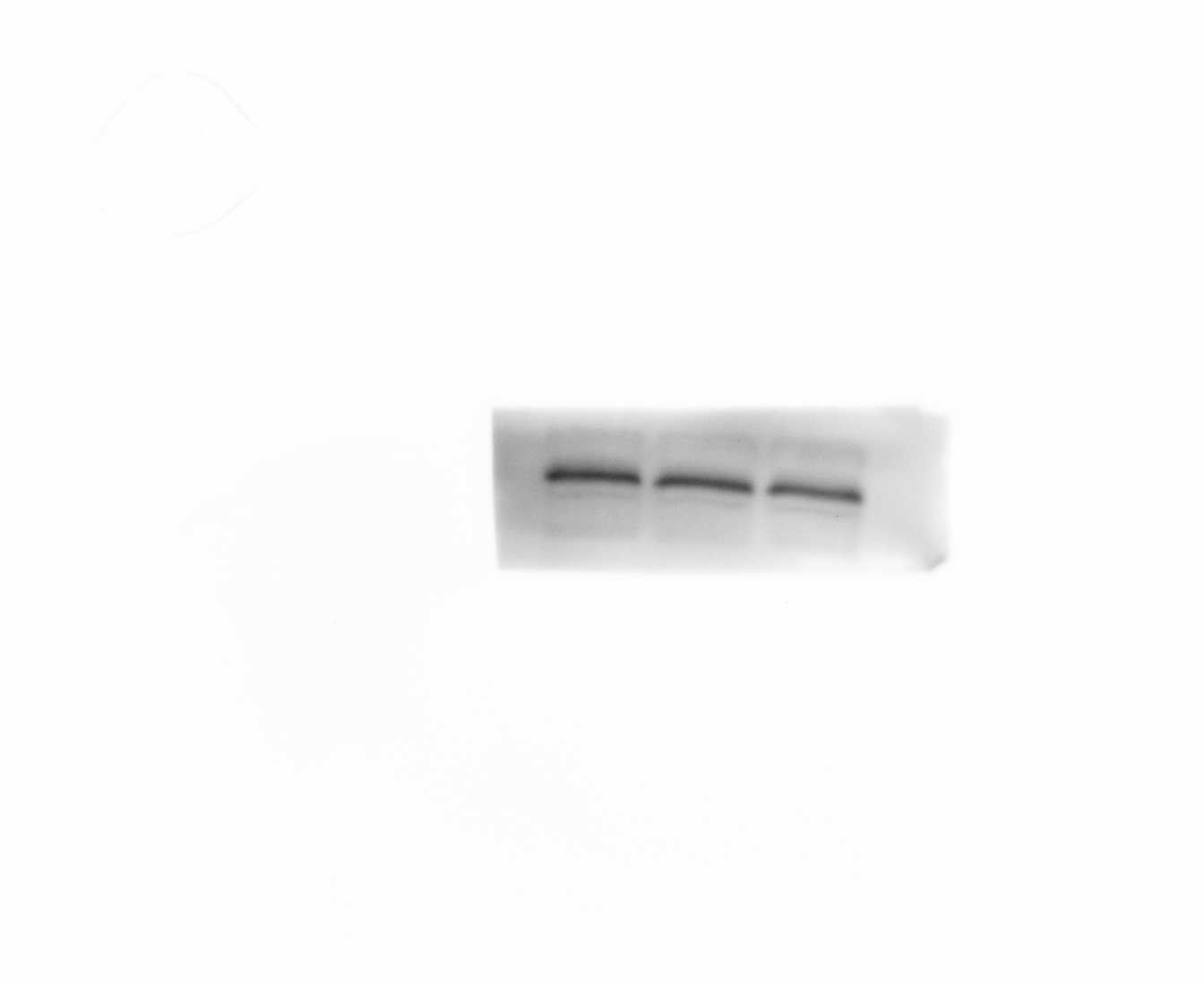

Supplement: Supplementary file 4 [file DataSheet4.ZIP › Western blot/Cyclin D1.tif]

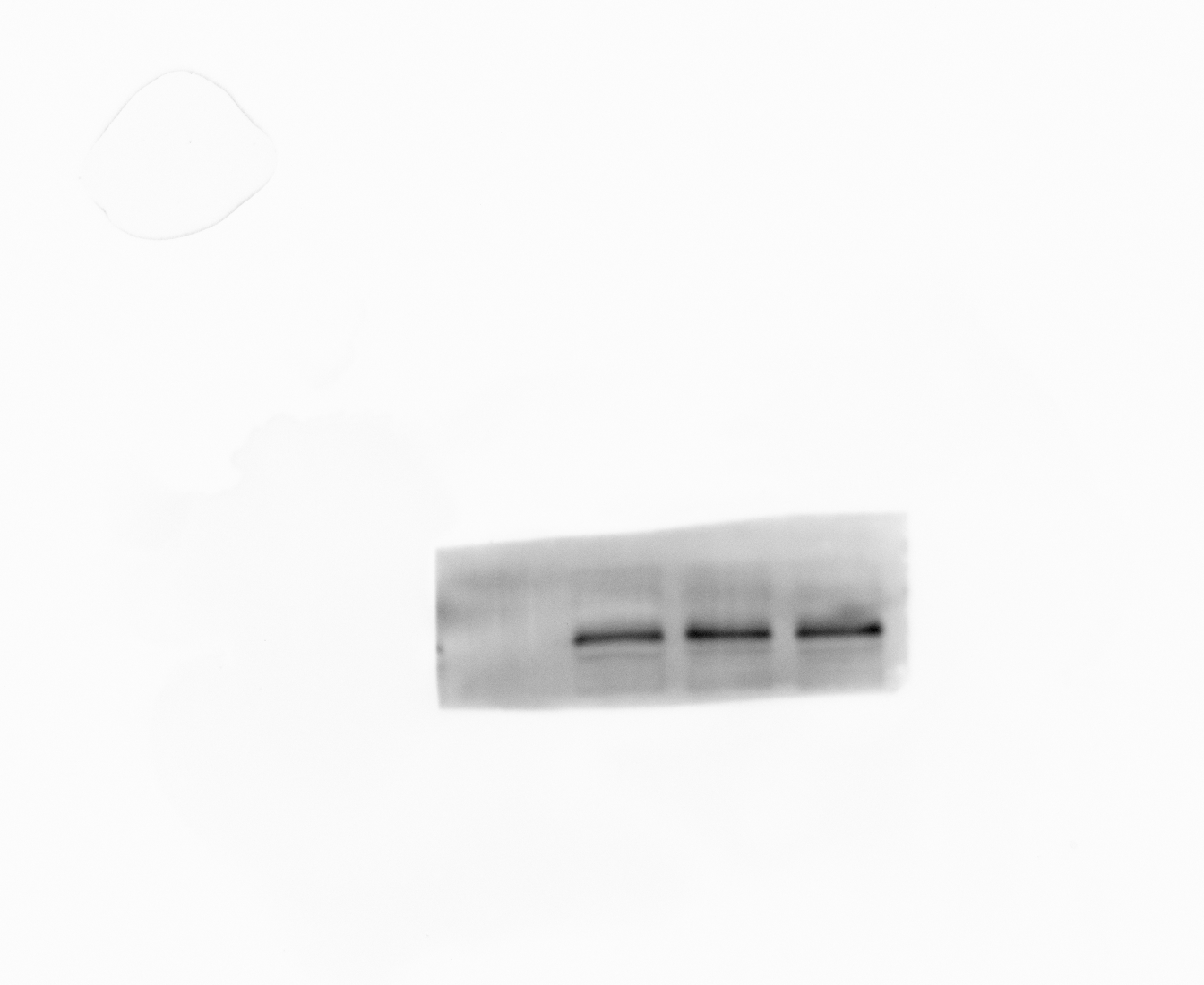

Supplement: Supplementary file 4 [file DataSheet4.ZIP › Western blot/GAPDH-Cyclin D1.tif]

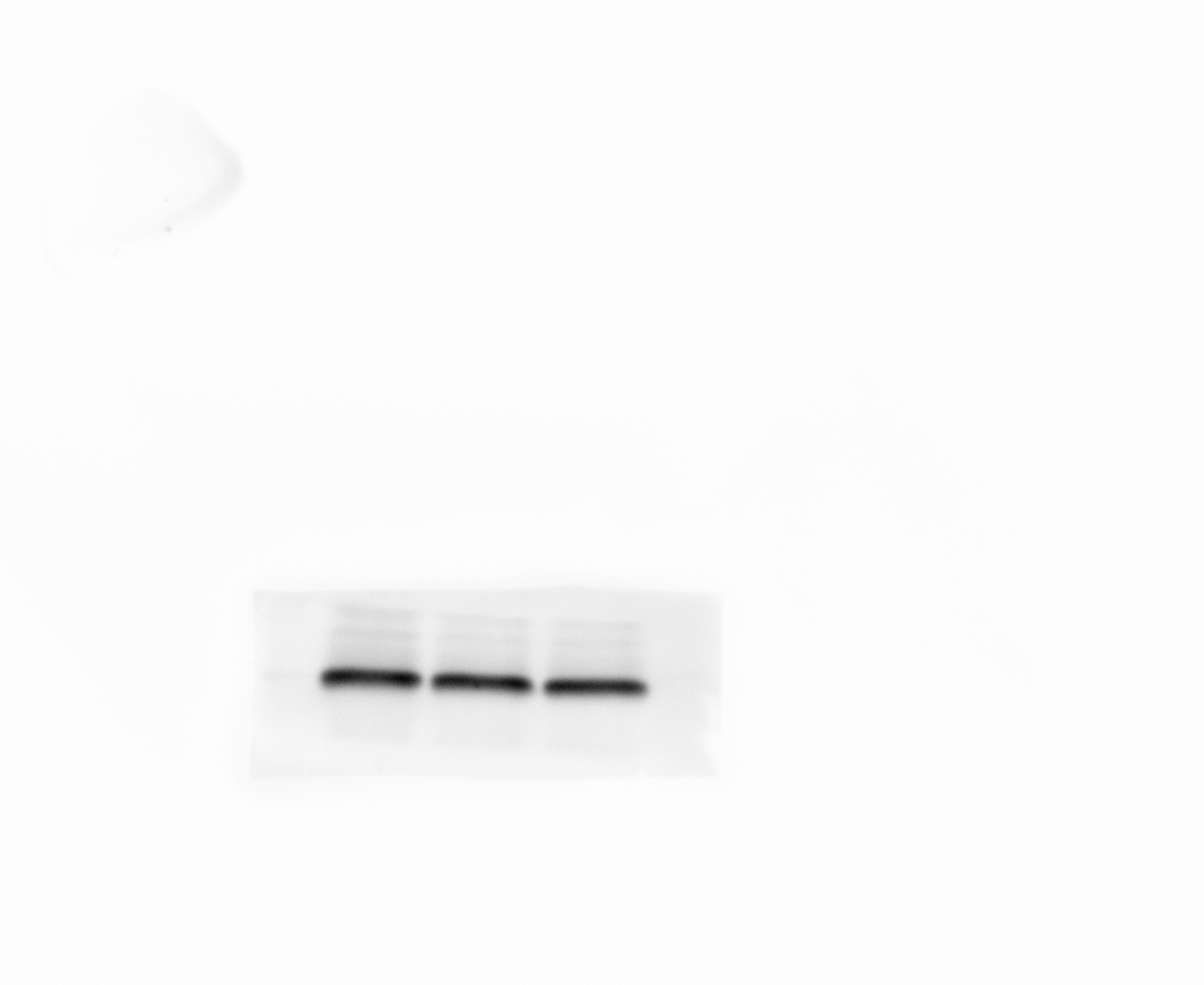

Supplement: Supplementary file 4 [file DataSheet4.ZIP › Western blot/GAPDH-MMP-11.tif]

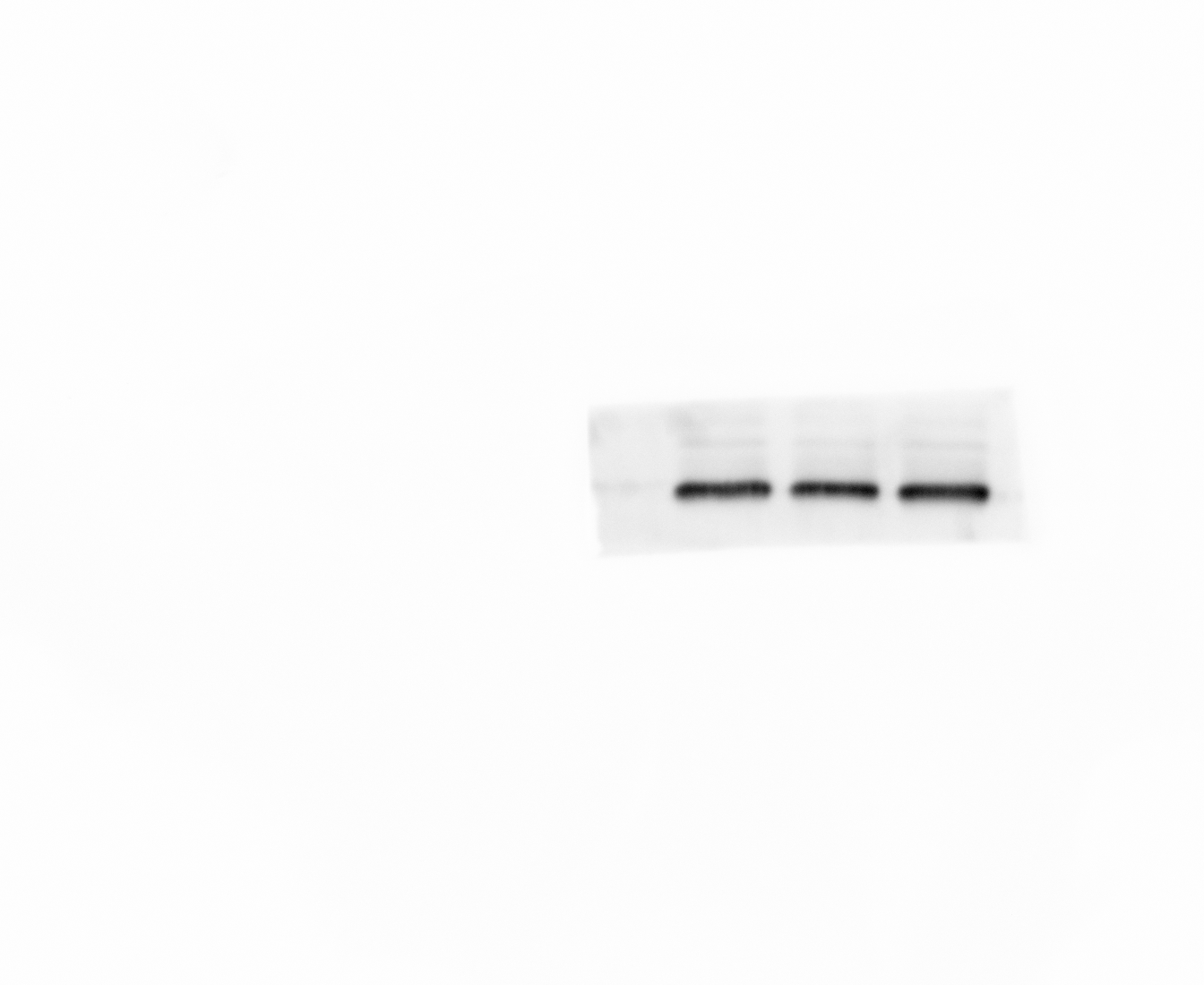

Supplement: Supplementary file 4 [file DataSheet4.ZIP › Western blot/GAPDH-PI3K-AKt-mTOR.tif]

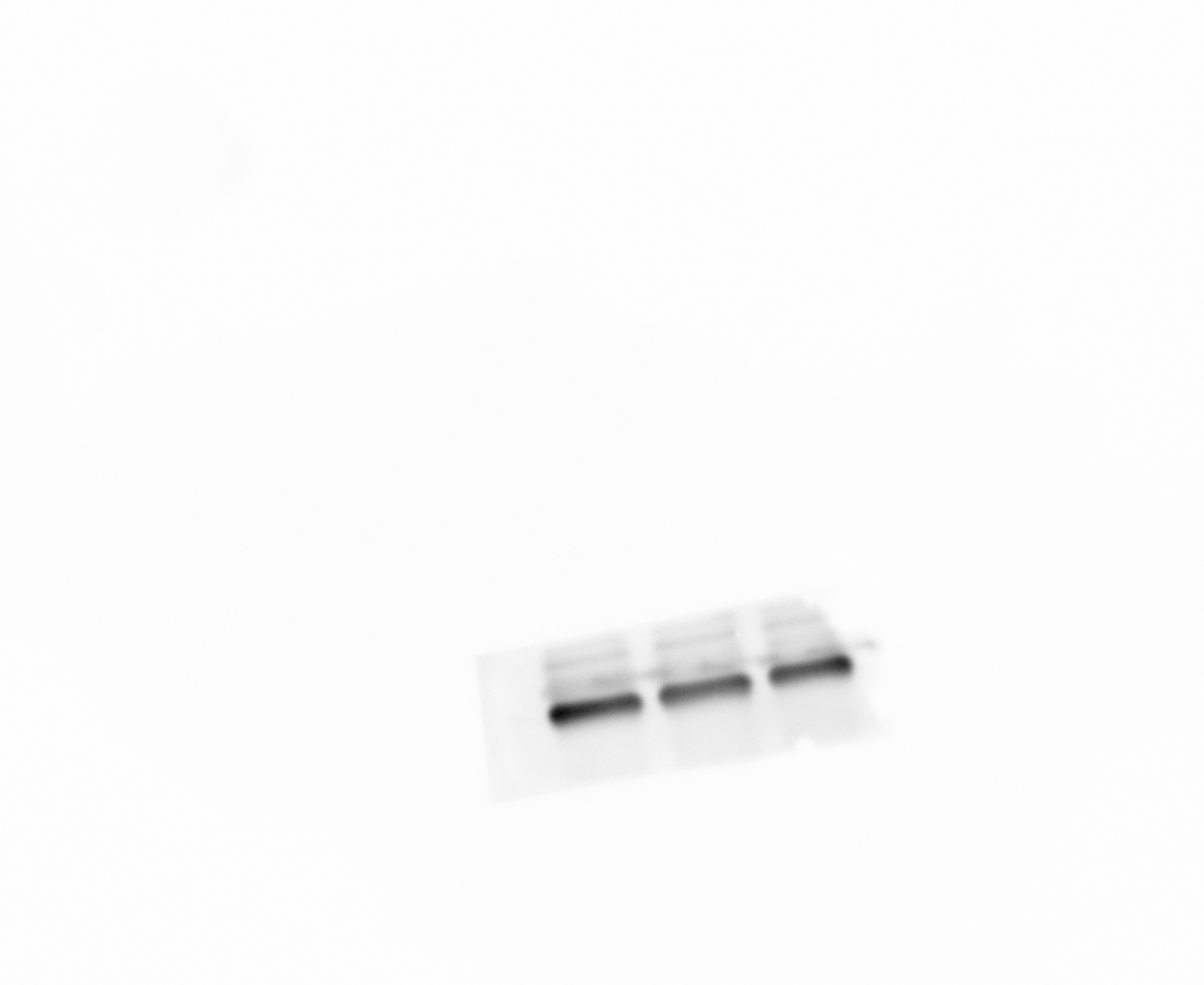

Supplement: Supplementary file 4 [file DataSheet4.ZIP › Western blot/MMP-11.tif]

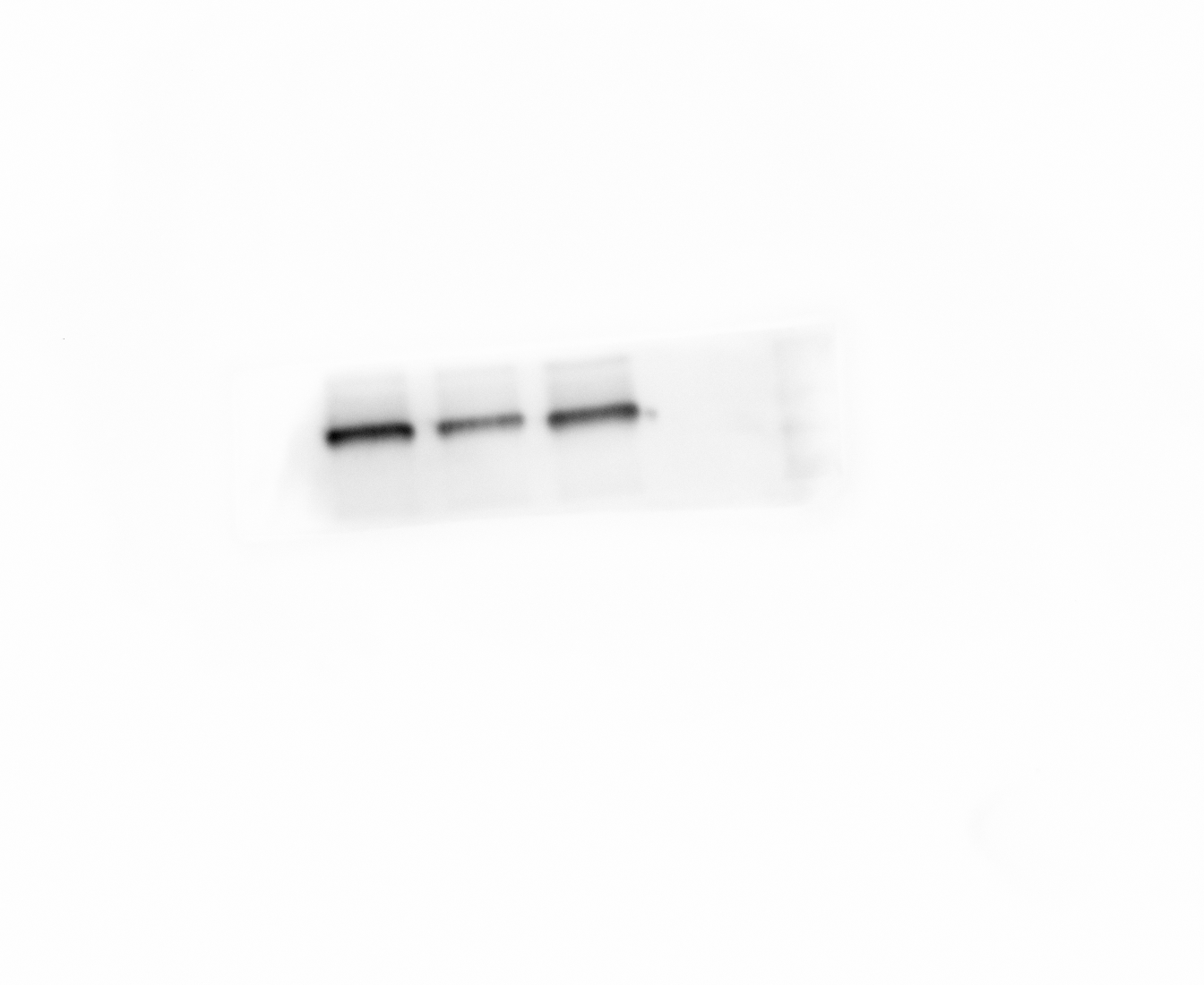

Supplement: Supplementary file 4 [file DataSheet4.ZIP › Western blot/mTOR.tif]

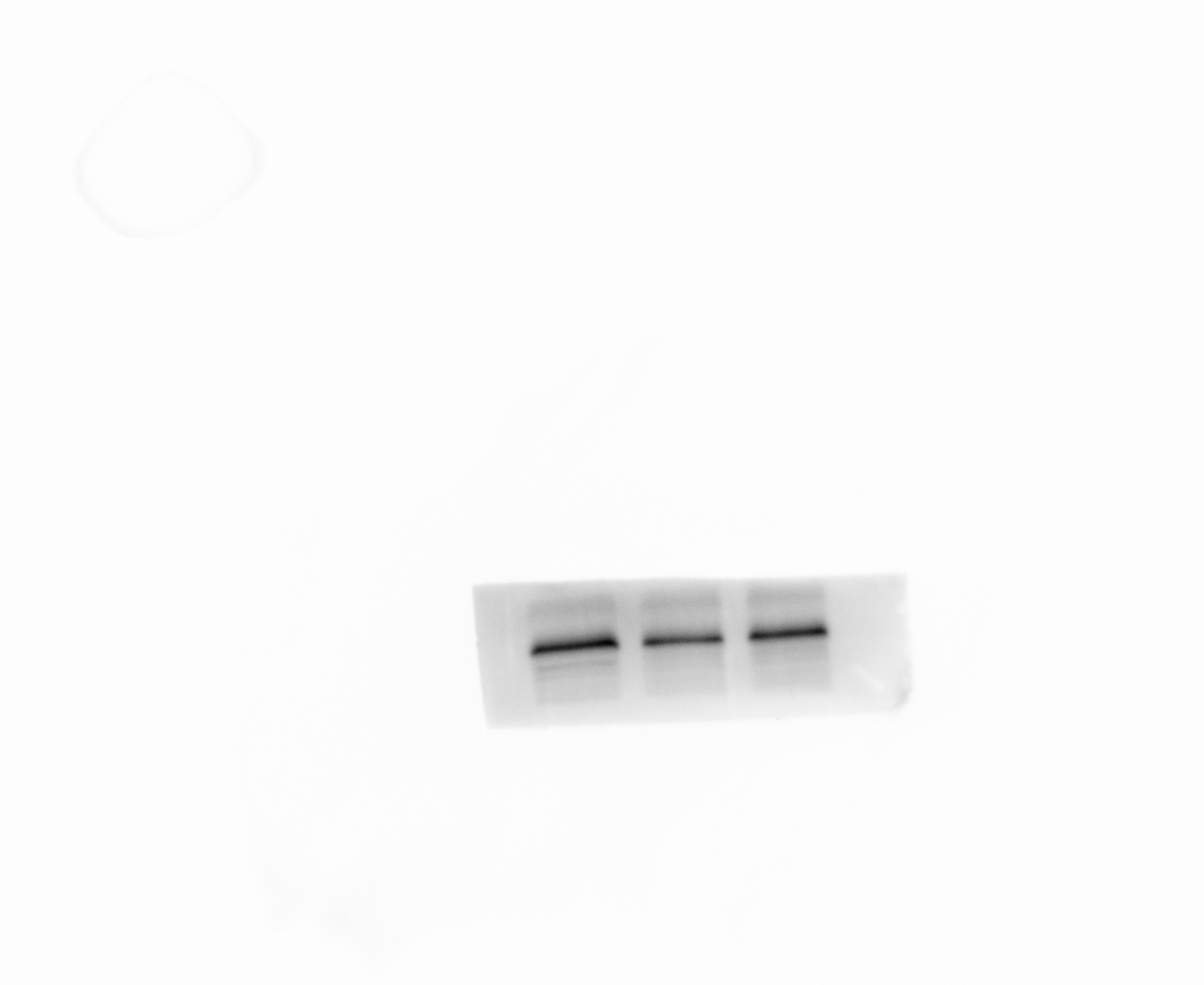

Supplement: Supplementary file 4 [file DataSheet4.ZIP › Western blot/PI3K.tif]

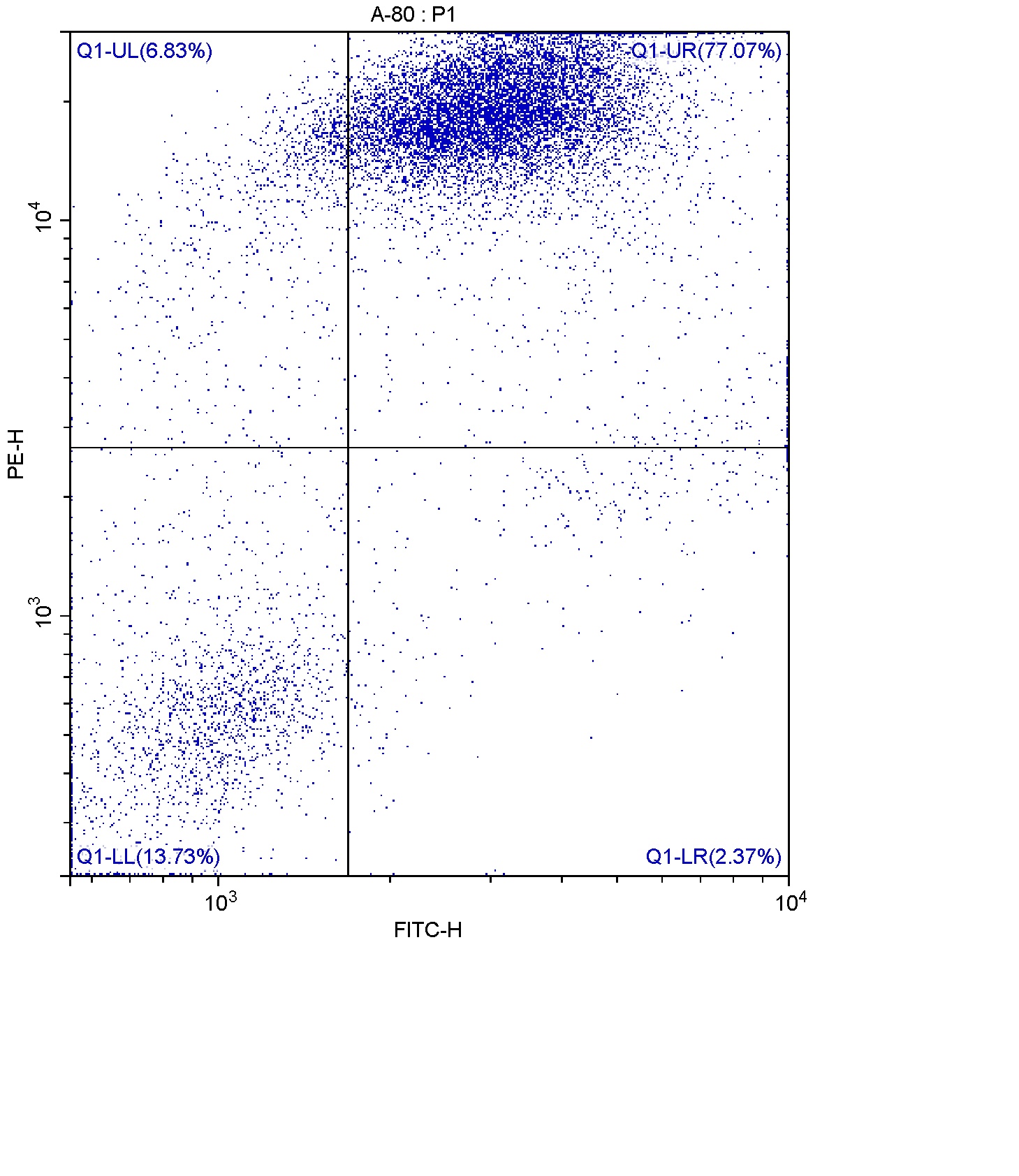

Supplement: Supplementary file 5 [file DataSheet1.ZIP › Flow Cytometry/metformin control group.bmp]

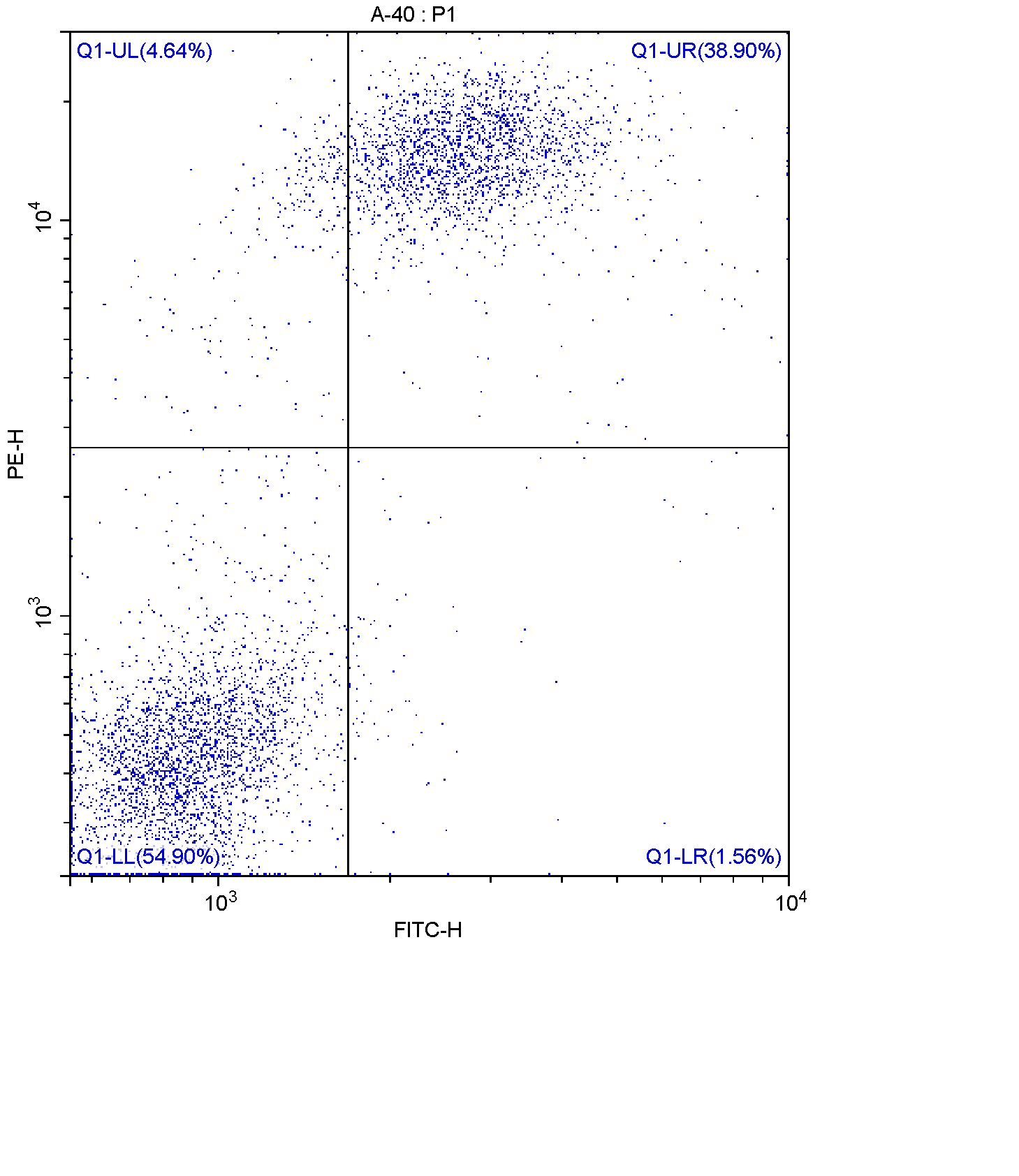

Supplement: Supplementary file 5 [file DataSheet1.ZIP › Flow Cytometry/Blank control group.bmp]

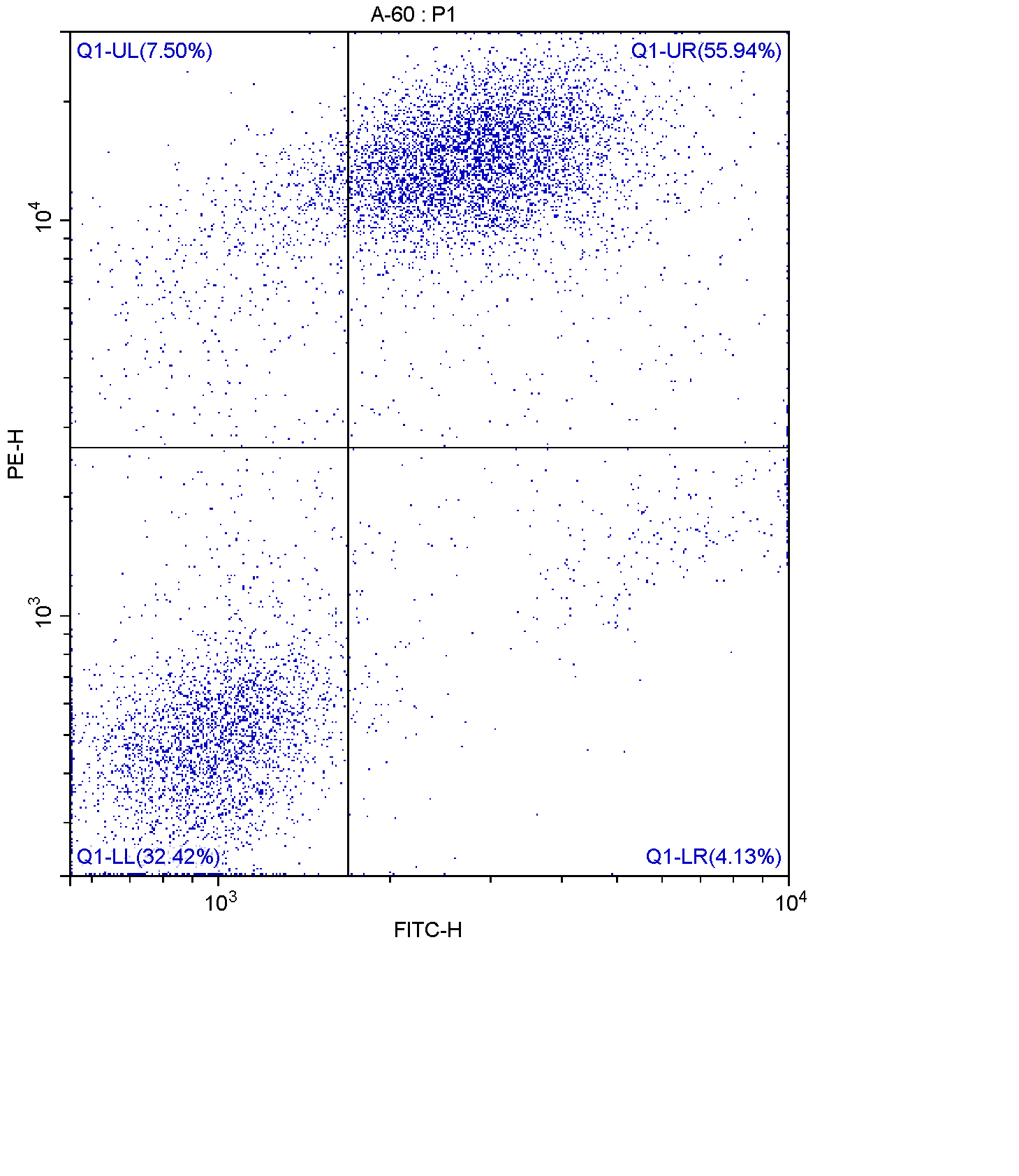

Supplement: Supplementary file 5 [file DataSheet1.ZIP › Flow Cytometry/Liraglutide group.bmp]

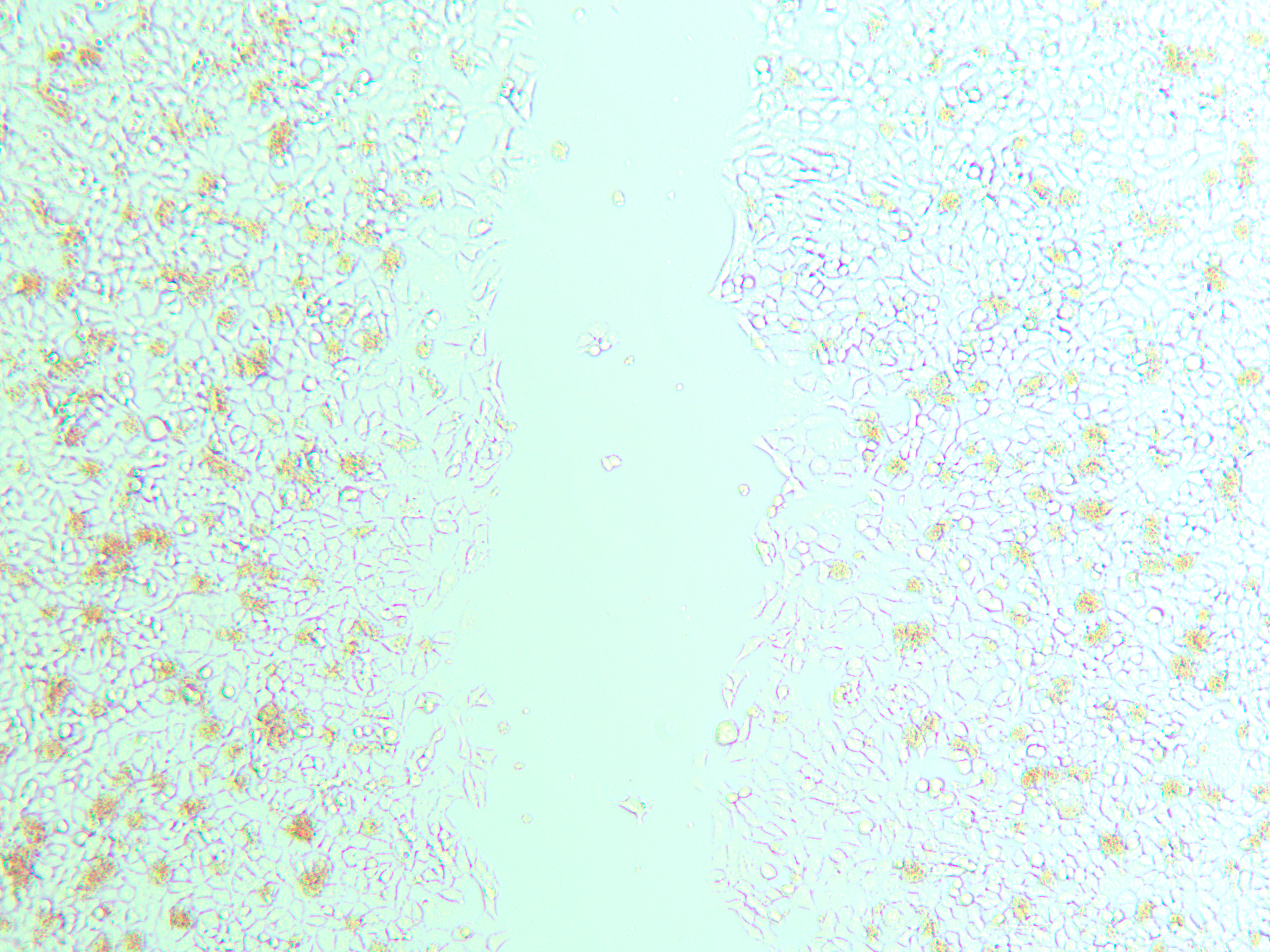

Supplement: Supplementary file 6 [file DataSheet10.ZIP › metformin control group 12h.bmp]

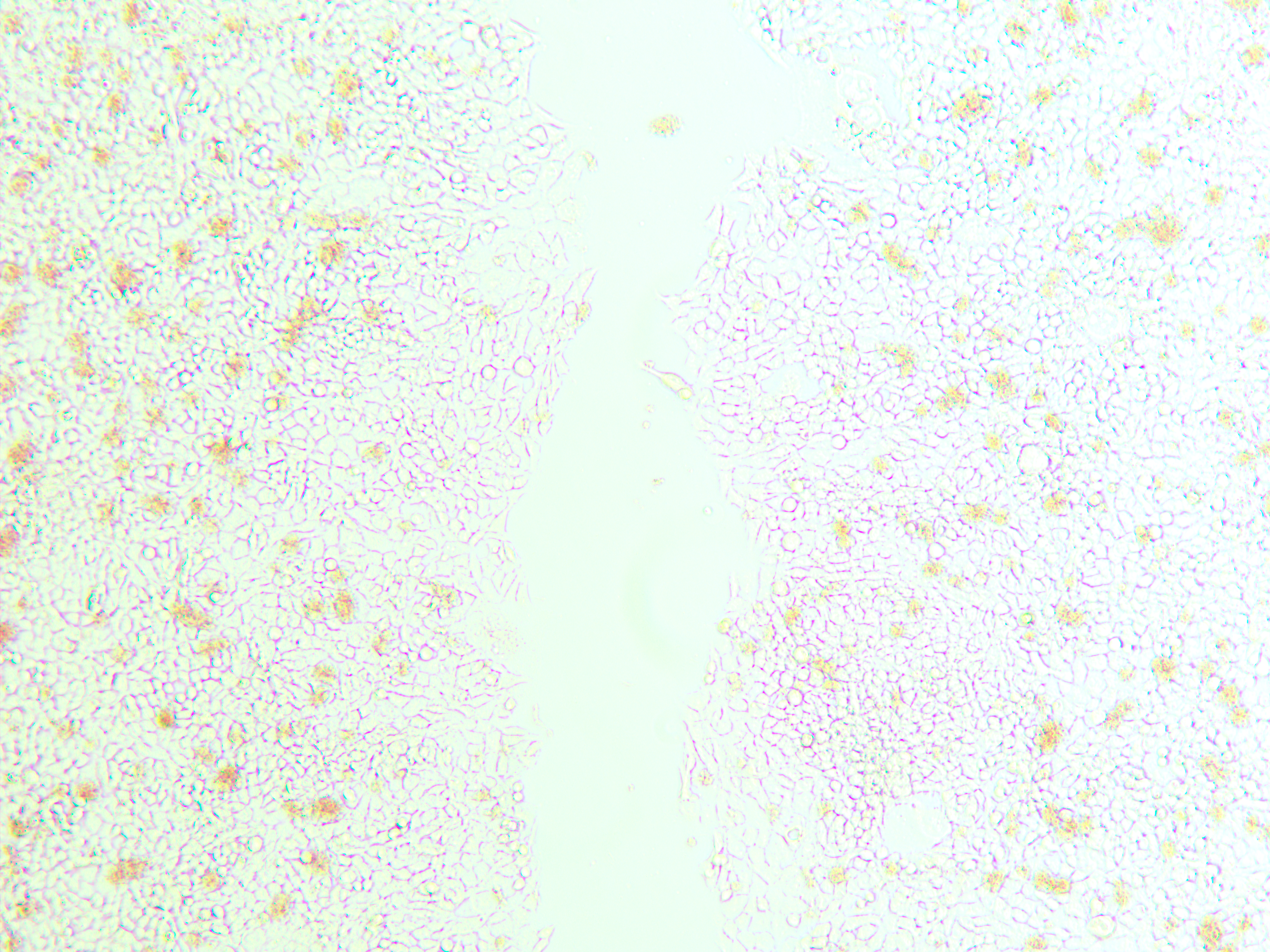

Supplement: Supplementary file 7 [file DataSheet6.ZIP › Blank control group 12h.bmp]

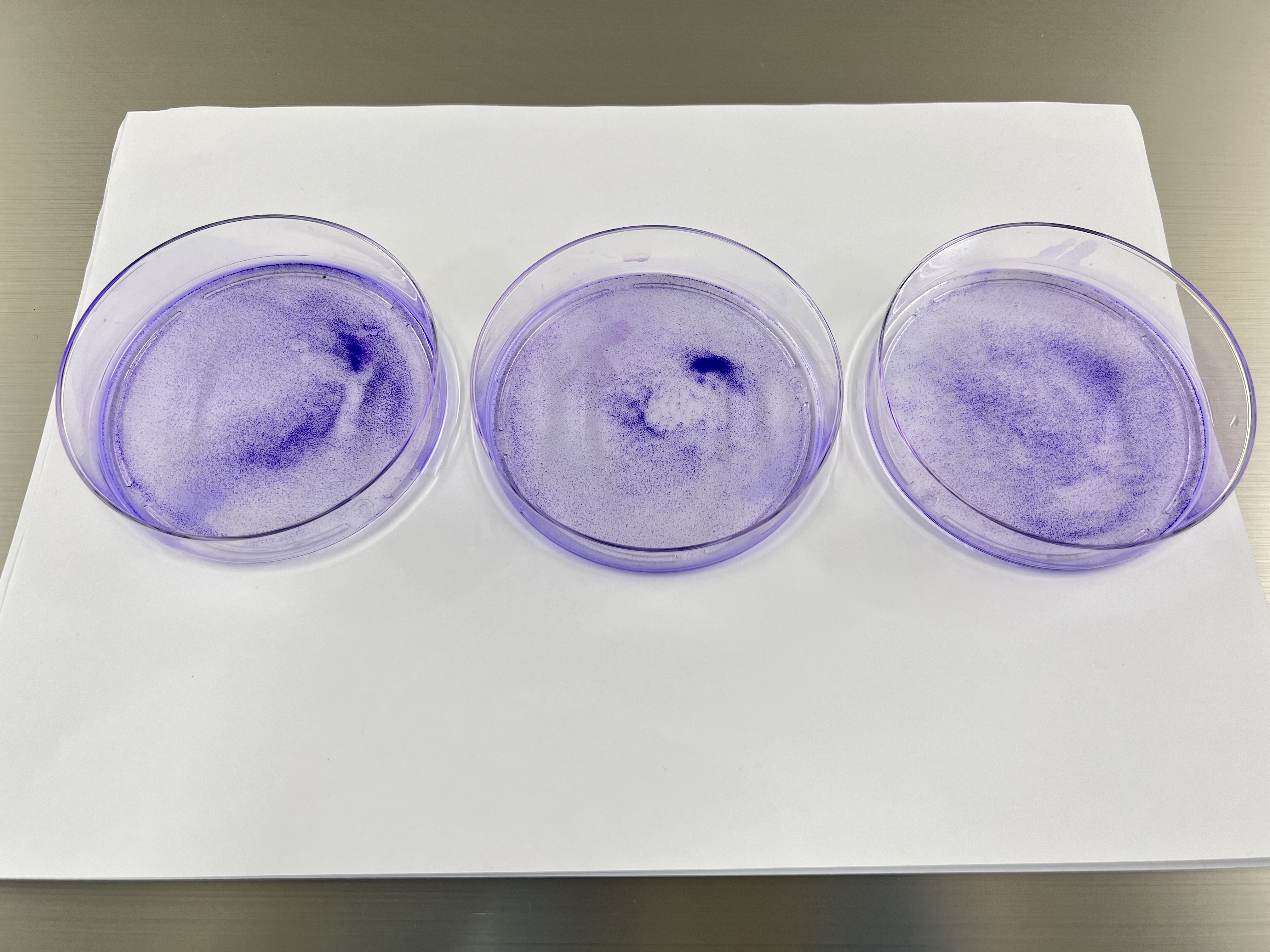

Supplement: Supplementary file 8 [file DataSheet2.ZIP › Plate clone/Blank control group metformin control group Liraglutide group.jpg]

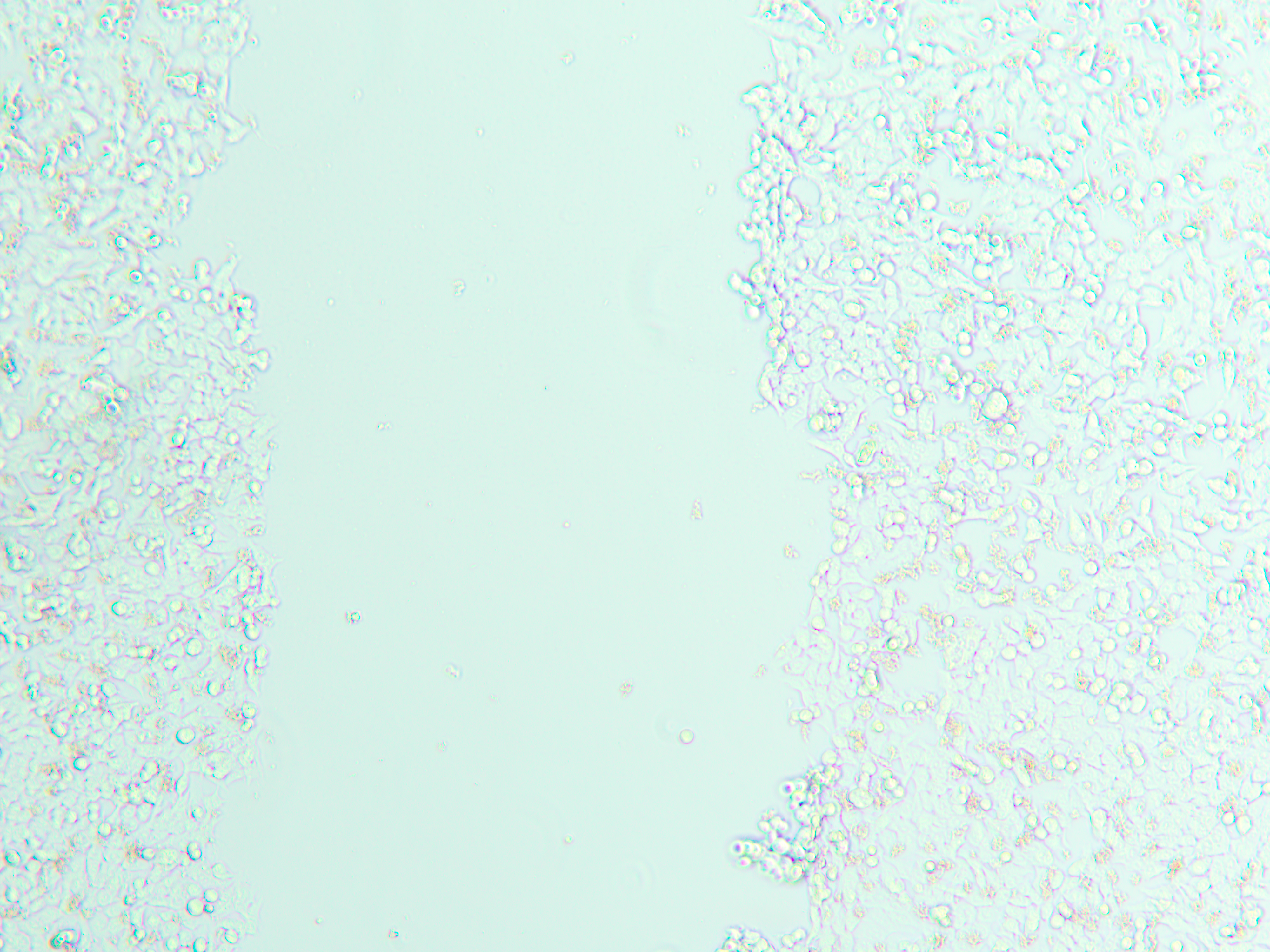

Supplement: Supplementary file 9 [file DataSheet5.ZIP › Blank control group 0h.bmp]

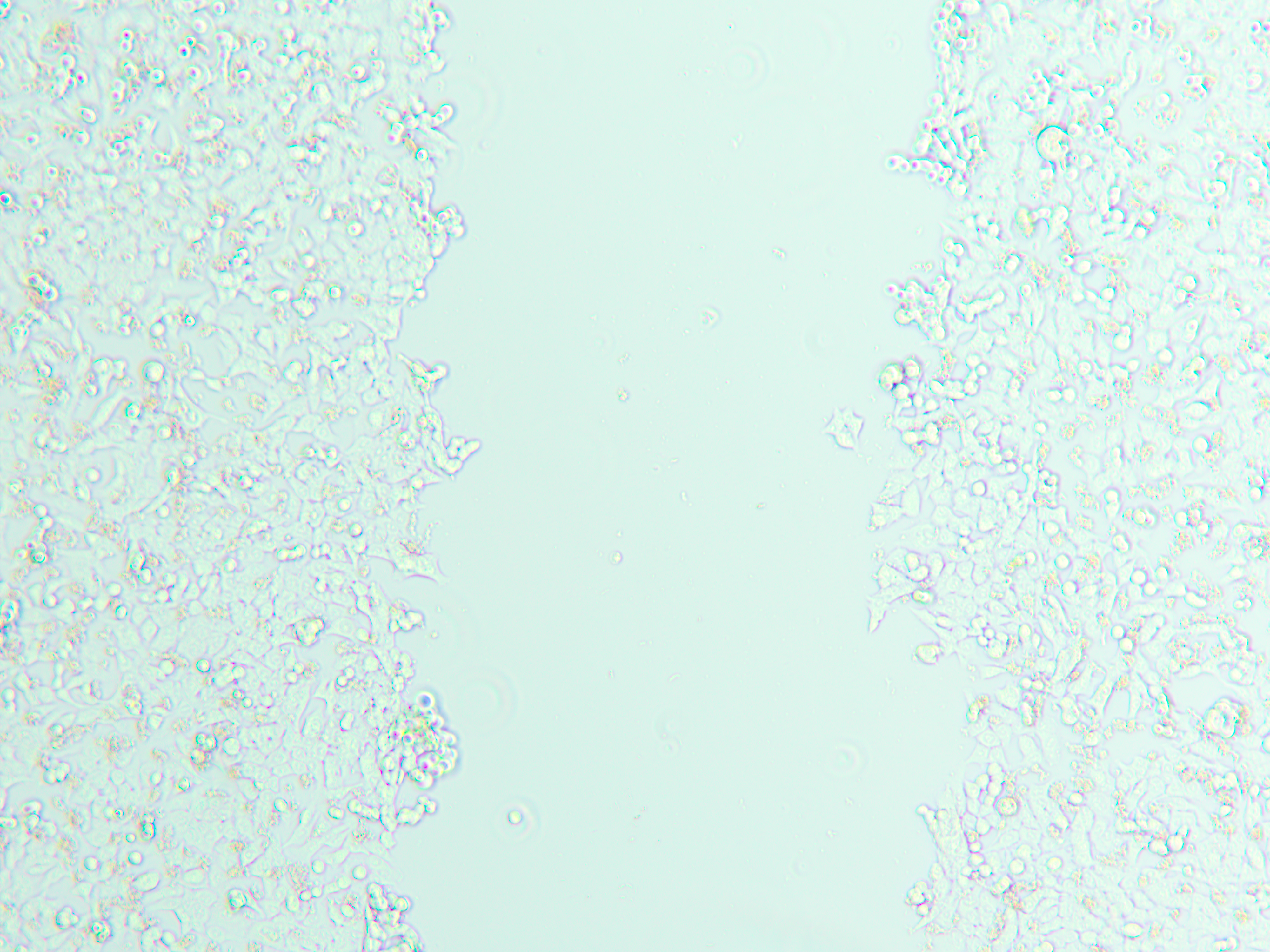

Supplement: Supplementary file 10 [file DataSheet7.ZIP › Liraglutide group 0h.bmp]
